# Dietary folic acid coordinates growth and lipid metabolism through gut microbial and one-carbon networks in juvenile mud crab (*Scylla paramamosain*)

**DOI:** 10.1016/j.aninu.2026.01.019

**Published:** 2026-07-07

**Authors:** Zheng Tang, Yao Deng, Shichao Xie, Wenhao Zhan, Hongyu Peng, Yinqiu Tian, Min Jin, Tiantian Xu, Xishuai Cui, Xiaoyue Li, Peng Sun, Qicun Zhou

**Affiliations:** Laboratory of Fish and Shellfish Nutrition, School of Marine Sciences, Ningbo University, Ningbo 315211, Zhejiang, China

**Keywords:** Folic acid, *Scylla paramamosain*, Lipid metabolism, One-carbon metabolism, Gut microbial

## Abstract

This study investigated the effects of dietary folic acid (FA) on growth performance, lipid metabolism, one-carbon metabolism, and gut microbiota of juvenile mud crab (*Scylla paramamosain*). Six isonitrogenous and isolipidic diets were formulated: one antibiotic basal diet control with 1% succinylsulfathiazole (0.00 + 1% SST), one basal diet control (0.00 mg/kg FA), and four diets with graded FA levels (1.37, 2.43, 4.26, and 9.84 mg/kg). A total of 180 juvenile crabs (initial body weight: 15.21 ± 0.10 g) were individually reared for 8 weeks. Compared with the 0.00 mg/kg FA and 0.00 + 1% SST treatments, dietary supplementation with 4.26 mg/kg FA improved final weight (FW), percent weight gain (PWG), specific growth rate (SGR), survival, and feed efficiency (FE) (*P* < 0.05). The 4.26 mg/kg FA also enhanced eicosapentaenoic acid (EPA) and docosahexaenoic acid (DHA) contents in hepatopancreas and muscle, whereas the 0.00 + 1% SST treatment significantly reduced total EPA + DHA contents (*P* < 0.05). Moreover, crabs fed 4.26 mg/kg FA exhibited decreased hemolymph aspartate aminotransferase (AST) and alanine aminotransferase (ALT) activities, significantly reduced malondialdehyde (MDA) concentrations in hemolymph and hepatopancreas, and significantly increased total superoxide dismutase (T-SOD) activity in the hepatopancreas (*P* < 0.05). Additionally, 4.26 mg/kg FA elevated S-adenosylmethionine (SAM) and S-adenosylhomocysteine (SAH) concentrations and upregulated the expression of *pcft*, *dhfr*, *shmt*, *mthfr*, and *mtr* in the hepatopancreas (*P* < 0.05). The 16S rRNA gene sequencing further revealed that 4.26 mg/kg FA increased the relative abundance of gram-negative and aerobic bacteria (*P* < 0.05), while reducing oxidative stress-tolerant, anaerobic, and potentially pathogenic bacteria in the intestine. These findings indicate that juvenile mud crab have a limited capacity for FA synthesis and require dietary supplementation. A dietary FA level of 4.26 mg/kg supported optimal growth and physiological functions, while two-slope broken-line regression of PWG against dietary FA levels estimated an optimal dietary requirement of 3.86 mg/kg for juvenile mud crab.

## 1 Introduction

Folic acid (FA), a water-soluble member of the B-vitamin family, is widely utilized as a nutritional supplement in both human foods and animal diets (Cheng et al., 2024). In vivo, FA is metabolically converted into tetrahydrofolate (THF), an active form that functions as a cofactor for one-carbon unit transfer. Through this process, FA plays essential roles in DNA synthesis and repair, cell proliferation, and amino acid metabolism (Wilt et al., 2021). In aquatic animals, appropriate dietary FA supplementation not only improves growth performance, but also enhances antioxidant defense and immune responses (Asaikkutti et al., 2016; NRC, 2011; Sesay et al., 2016). To date, FA requirements have been established for several species, including flathead grey mullet (*Mugil cephalus*, 30–50 mg/kg), giant tiger shrimp (*Penaeus monodon*, 2 mg/kg), and Chinese mitten crab (*Eriocheir sinensis*, 2.29–2.90 mg/kg) (Badran and Ali, 2021; Shiau and Huang, 2001; Wei et al., 2016). However, substantial interspecific variation in FA requirements has been reported, which may be influenced by species-specific physiology, developmental stage, diet composition, environmental factors, and methodological differences.

Beyond its classical role in nucleotide and amino acid metabolism, FA has been increasingly recognized as a key regulator of lipid metabolism (Hirsch et al., 2005). For example, FA supplementation mitigated hepatic steatosis in mice fed high-fat diets, and improved plasma lipid profiles while reducing cerebrovascular disease risk in humans (Fogacci et al., 2024; Sid et al., 2015). These effects are largely mediated through one-carbon metabolism, in which one-carbon units (methyl, methylene, and formyl groups) bind covalently to THF and participate in the methionine cycle, transsulfuration pathway, and purine/pyrimidine biosynthesis (Guéant et al., 2020; Maclean et al., 2021). Intermediates such as S-adenosylmethionine (SAM) and S-adenosylhomocysteine (SAH) are thought to be central regulators linking FA metabolism to lipid homeostasis (da Silva et al., 2014). Although the mechanisms underlying FA-mediated lipid regulation remain largely unexplored in aquatic animals, available evidence provides a theoretical framework for further investigations.

The gut microbiota is another key factor influencing host nutrition, growth, and immunity in aquatic animals (Shi et al., 2025). Dietary additives are increasingly recognized for their ability to modulate microbial composition and function (Gao et al., 2025; Li et al., 2024). Research demonstrates that FA can enhance the population of advantageous gut microbiota, improve intestinal epithelial integrity, optimize bacterial metabolic activities, and boost the generation of short-chain fatty acids (SCFAs) (Bai et al., 2021; Mardinoglu et al., 2018; Sun et al., 2025). In addition, certain microbes, including *Lactobacillus*, *Bifidobacterium*, and certain types of *Streptococcus*, are capable of synthesizing FA (Khedr et al., 2023; Padalino et al., 2012). Comparative analyses reveal distinct compositional divergence in gut microbiota between terrestrial and aquatic species (Cannicci et al., 2020). Notably, the potential regulatory effects of FA supplementation on microbial communities in mud crab (*Scylla paramamosain*) remain largely unexplored, highlighting a critical knowledge gap that warrants systematic investigation.

The mud crab, a commercially valuable decapod species, is extensively farmed in China's coastal regions (Zhao et al., 2025). With declining wild stocks, aquaculture has become the primary means of supply (Wu et al., 2019). However, the lack of a comprehensive nutritional requirement database for mud crabs often leads to issues of under- or over-nutrition, ultimately compromising growth and health (Xie et al., 2025; Zhao et al., 2023). Therefore, the present study aims to evaluate the effects of dietary FA on growth performance, lipid metabolism, one-carbon metabolism, and gut microbiota of juvenile mud crab. The findings of this study are expected to enrich the nutritional database for mud crabs and provide a scientific basis for optimizing diet formulations and elucidating FA-associated metabolic pathways in crustaceans.

## 2 Materials and methods

### 2.1 Animal ethics statement

The current research adhered strictly to Ningbo University's institutional animal care and use guidelines (No. SYXK20190005). Prior to implementation, the mud crab experimentation protocol received formal ethical approval from Ningbo University's Animal Ethics Review Committee.

### 2.2 Experimental diet formulation and preparation methods

Six isonitrogenous and isolipidic experimental diets (approximately 42% crude protein and 9% crude lipid) were formulated. According to the experimental design, varying levels of FA were incorporated into the vitamin premix of each treatment. To examine whether mud crabs possess the ability to synthesize FA endogenously, the FA antagonist succinylsulfathiazole (SST) was included in the first treatment. High-performance liquid chromatography (HPLC; Shimadzu LC-10ATVP, Shimadzu Corporation, Kyoto, Japan) analysis determined the actual FA concentrations in the diets to be 0.00, 0.00, 1.37, 2.43, 4.26, and 9.84 mg/kg, respectively, which were consistent with the intended formulation. The detailed ingredient composition and proximate analysis of the diets are presented in Table 1.

**Table 1:** The detailed ingredient composition and proximate analysis of the diets (dry matter basis).

Items	Dietary folic acid levels, mg/kg					
	0.00 + 1% SST	0.00	1.37	2.43	4.26	9.84
**Ingredients**^1^**, g/kg**						
Peru fish meal	300.00	300.00	300.00	300.00	300.00	300.00
Poultry by product meal	30.00	30.00	30.00	30.00	30.00	30.00
Soybean meal	180.00	180.00	180.00	180.00	180.00	180.00
Soybean protein concentrate	80.00	80.00	80.00	80.00	80.00	80.00
Peanut meal	30.00	30.00	30.00	30.00	30.00	30.00
Krill meal	30.00	30.00	30.00	30.00	30.00	30.00
Yeast extract	30.00	30.00	30.00	30.00	30.00	30.00
Wheat flour	217.50	217.50	217.50	217.50	217.50	217.50
Fish oil	12.00	12.00	12.00	12.00	12.00	12.00
Soybean oil	12.00	12.00	12.00	12.00	12.00	12.00
Soy lecithin	10.00	10.00	10.00	10.00	10.00	10.00
Cholesterol	5.00	5.00	5.00	5.00	5.00	5.00
Vitamin premix^2^	5.00	5.00	5.00	5.00	5.00	5.00
Mineral premix^3^	10.00	10.00	10.00	10.00	10.00	10.00
Ca(H_2_PO_4_)_2_	20.00	20.00	20.00	20.00	20.00	20.00
Choline chloride	3.00	3.00	3.00	3.00	3.00	3.00
Sodium alginate	20.00	20.00	20.00	20.00	20.00	20.00
2, 6-Di-tert-butylhydroxytoluene	2.00	2.00	2.00	2.00	2.00	2.00
Cellulose	0.50	0.50	0.50	0.50	0.50	0.50
Lutein	3.00	3.00	3.00	3.00	3.00	3.00
Total	1000.00	1000.00	1000.00	1000.00	1000.00	1000.00
Folic acid^4^, mg/kg	0.00	0.00	1.00	2.00	4.00	8.00
Succinylsulfathiazole (SST)^5^	10.00	0.00	0.00	0.00	0.00	0.00
**Proximate composition**^6^**, %**						
Crude protein	42.84	42.70	42.71	42.74	42.85	42.89
Crude lipid	9.86	9.67	9.58	9.83	9.96	9.63
Dry matter	88.92	88.76	89.29	89.40	88.79	89.45
Organic matter	79.62	79.34	79.86	80.07	79.42	80.03
Gross energy, MJ/kg	17.66	17.62	17.58	17.65	17.71	17.63
Folic acid, mg/kg	0.00	0.00	1.37	2.43	4.26	9.84

1 All ingredients, except for vitamin premix, folic acid and succinylsulfathiazole, were purchased from Tech-Bank Feed Co., Ltd., Ningbo, Zhejiang, China.

2 Vitamin premix (g/kg premix): retinyl acetate, 1.2121; cholecalciferol, 1.2000; all-rac-α-tocopherol, 20.0000; menadione, 9.0909; thiamine, 10.8696; riboflavin, 7.5000; ascorbic acid, 30.0000; pyridoxine hydrochloride, 12.1212; cyanocobalamin, 2.0000; biotin, 12.5000; nicotinic acid, 40.4040; D-Ca pantothenate, 16.1290; inositol, 204.0817; cellulose, 632.8915. Purchased from DSM, Vitamin Shanghai Co., Ltd. (Shanghai, China). The vitamin premix does not contain folic acid.

3 Mineral premix (g/kg premix): FeC_6_H_5_O_7_, 4.57; ZnSO_4_·7H_2_O, 9.43; MnSO_4_·H_2_O (99%), 4.14; CuSO_4_·5H_2_O (99%), 6.61; MgSO_4_·7H_2_O (99%), 238.97; KH_2_PO_4_, 233.2; NaH_2_PO_4_, 137.03; C_6_H_10_CaO_6_·5H_2_O (98%), 34.09; CoCl_2_·6H_2_O (99%), 1.36.

4 The folic acid supplementation levels for the different treatments were achieved by adjusting its amount added to the vitamin premix. Purchased from DSM, Vitamin Shanghai Co., Ltd. (Shanghai, China).

5 Purchased from Shanghai Aladdin Biochemical Technology Co., Ltd. (Shanghai, China).

6 All the values were analyzed.

The preparation and storage procedures of the experimental diets followed the methods previously established in the laboratory (Wang et al., 2020). A standardized particle size distribution was achieved by subjecting all feed components to mechanical pulverization followed by size fractionation using an 80-mesh sieve. Each component was accurately weighed according to the formulated proportions. The vitamin premix (specific to each treatment), mineral premix, other trace additives, and major dietary ingredients were thoroughly blended using a stepwise scaling-up method, after which the lipid components were added. The pre-mixed materials were then transferred to a Hobart-type mixer (B20–W, Guangdong Henglian Food Machinery Co., Ltd., Guangzhou, Guangdong, China), where distilled water was gradually incorporated during mixing. The homogenized mixture was processed into pellets of two sizes (3.0 and 5.0 mm in diameter) using a cold extruder (F-26) and a pelletizer (G-250) from South China University of Technology (Guangzhou, Guangdong, China). Finally, all diets were oven-dried at 45 °C for 10 h, vacuum-sealed in light-proof plastic bags, and stored at −20 °C until the feeding trial commenced.

### 2.3 Feeding trial and culture management

The feeding trial was conducted at the crab rearing facility of Meishan campus aquaculture base of Ningbo University (Ningbo, Zhejiang, China). All experimental crabs were obtained from a commercial hatchery (Taizhou, Zhejiang, China). In accordance with standard operating procedures for aquaculture facilities, the recirculating aquaculture system (RAS) was thoroughly sanitized prior to the commencement of the experiment, including mechanical cleaning and chemical disinfection (Tang et al., 2024). After a two-week acclimation period on a commercial diet, 180 healthy crabs with similar initial body weight (15.21 ± 0.10 g), intact appendages, and no visible signs of disease were randomly allocated to six dietary treatments. Each treatment included three replicates, with ten crabs per replicate, and the feeding trial lasted for eight weeks (56 d in total). To prevent aggressive interactions, each crab was maintained individually in a glass compartment (48.3 cm × 28.4 cm × 38.0 cm).

The crabs received formulated diets at 08:00 and 18:00 daily, with the ration quantity maintained at 4% of their individual body weight throughout the experimental period. The daily feeding amount was dynamically adjusted according to the crabs’ feeding status (observed feeding speed and leftover feed checks) and was appropriately increased after molting based on the recovery condition. Mortality and molting events were recorded daily. Uneaten diets and feces were promptly removed by siphoning. All dead crabs and molts were promptly scooped out using a dedicated net. The RAS implemented a partial water exchange method, with two-thirds of the total seawater volume being systematically replaced at 48 h intervals throughout the experimental period. Water quality was carefully maintained throughout the experiment, with dissolved oxygen kept above 6.2 mg/L, temperature controlled between 26.0 and 26.8 °C, and salinity maintained within 23.2 to 25.1 g/kg.

### 2.4 Sample collection

At the end of the feeding trial, all crabs had completed at least one molting cycle. Prior to dissection, the number of surviving crabs in each treatment was recorded following the procedure described by Xie et al. (2024). Individual body weights were measured to calculate the percent weight gain (PWG), specific growth rate (SGR), and feed efficiency (FE). For each replication of every treatment, four crabs were randomly selected and anesthetized on ice. Hemolymph was immediately collected from the pericardial cavity using 1-mL sterile syringes and transferred into 2-mL centrifuge tubes. Samples were maintained at 4 °C for 24 h, then centrifuged at 1229 × *g* for 10 min at 4 °C using a refrigerated centrifuge (5418R, Eppendorf AG, Hamburg, Germany). The resulting supernatant was aliquoted into 200-μL PCR tubes, labeled according to treatment, and stored at −80 °C for subsequent biochemical analyses. Intestinal tissues were carefully excised using sterile forceps, rinsed gently with phosphate-buffered saline (PBS; pH 7.4), transferred to sterile cryovials, and flash-frozen in liquid nitrogen for gut microbiota analysis. Hepatopancreas samples were similarly dissected on ice, and approximately 2 to 3 mm tissue fragments were transferred into 1.5-mL centrifuge tubes containing RNAlater (Takara Biomedical Technology Co., Ltd., Beijing, China). Following fixation for 12 h at 4 °C, the specimens were preserved in a −80 °C freezer to facilitate later RNA isolation and transcriptional profiling.

Additionally, two crabs per replicate that were not used for hemolymph collection were processed to obtain hepatopancreas samples (2–3 mm fragments), which were immediately immersed in 4% paraformaldehyde solution for histological examination. The remaining hepatopancreas tissues were aliquoted into 5-mL centrifuge tubes and cryopreserved at −80 °C to maintain enzymatic integrity for subsequent biochemical analyses.

### 2.5 Biochemical analysis

#### 2.5.1 Proximate composition

Dietary components were assayed according to AOAC (2006) prescribed techniques, ensuring methodological traceability. Moisture content quantification was performed through thermal dehydration in an oven (DHG-9240A, Shanghai Jinghong Laboratory Equipment Co., Ltd., Shanghai, China) maintained at 105 °C until stable mass was attained, as per the established protocol (method 934.01). The dry matter content was subsequently calculated as the difference between 100% and the measured moisture percentage. The crude protein content was determined using a rapid nitrogen/protein analyzer (FP-528, Leco Corporation, St. Joseph, MI, USA) based on the Dumas combustion method (method 968.06). The crude lipid content was determined using a Soxhlet apparatus (SX-360, Tecator AB, Höganäs, Skåne County, Sweden) through petroleum ether extraction (method 2000.03). Ash content was determined by first carbonizing the samples on an electric hot plate until smoke-free, followed by incineration in a muffle furnace (SX-2410, Shanghai Experimental Electric Furnace Co., Ltd., Shanghai, China) at 550 °C for 8 h (method 942.05). Organic matter content was determined by subtracting the ash weight from the total dry matter weight (Hasanthi et al., 2025). In an oxygen bomb calorimeter (Parr 6200 Isoperibol Calorimeter, Parr Instrument Company, Moline, IL, USA), the diet samples were combusted in pure oxygen, and the resulting temperature rise is used to calculate its gross energy content (ISO, 1998).

#### 2.5.2 Folic acid content in diets

The actual FA concentrations in the experimental diets were determined using high performance liquid chromatography (HPLC) following the Chinese agricultural industry standard NY/T 2895-2016 (Ministry of Agriculture of the People's Republic of China, 2016). Dietary FA was extracted with a sodium carbonate solution, purified using anion-exchange solid-phase extraction, and subsequently separated and quantified by ultraviolet (UV) detection on an HPLC system (1260 Infinity II, Agilent Technologies, Inc., Santa Clara, CA, USA). The FA concentrations were calculated based on the corresponding peak areas obtained from the chromatograms.

#### 2.5.3 Fatty acids in hepatopancreas and muscle

One mL of 1 mg/mL methyl tricosanoate standard solution (Sigma Co., St. Louis, MI, USA) was accurately measured and transferred into glass tubes equipped with polytetrafluoroethylene (PTFE) gasket sealing caps. The solution was dried under a gentle stream of nitrogen using a nitrogen blowing instrument (Miulab NDK200, Hangzhou MIU Instruments Co., Ltd., Hangzhou, Zhejiang, China). To the glass tubes, 100 mg lyophilized sample powder and 3 mL of 0.25 mg/mL 2,6-di-tert-butylhydroxytoluene (BHT; Biochemical Technology Co., Ltd., Shanghai, China) solution were added. The tubes were treated with an ultrasonic cleaner (SB-4200D, Ningbo Scientz Biotechnology Co., Ltd., Ningbo, Zhejiang, China) for 10 min to enhance extraction. Subsequently, the tubes were incubated in a water bath at 80 °C for 4 h, with vortexing performed every 20 min to ensure thorough mixing. After incubation, the tubes were allowed to cool to room temperature naturally. To each tube, 1 mL of HPLC-grade n-hexane (Sigma Co., St. Louis, MI, USA) and 1 mL of distilled water were added, followed by vortex mixing and centrifugation for 1 min. The upper organic phase was collected using 1-mL syringes and filtered through 0.22-μm organic-phase ultrafilter membranes into sample vials. This filtration step was repeated once to ensure clarity of the extract. Finally, the filtered extracts were dried under a nitrogen stream.

Prior to injection, an appropriate gas chromatography column should be selected based on fatty acid properties, with parameters such as column length, internal diameter, and stationary phase coating being considered. The mass spectrometer settings, including ionization mode (typically electron impact [EI]), are configured to optimize detection. The pretreated samples are injected into the gas chromatography-mass spectrometry (GC–MS) system (7890B-5977A, Agilent Technologies, Inc., Santa Clara, CA, USA) for separation and detection. Fatty acid components are identified through analysis of the recorded mass spectra. The concentrations of individual fatty acids are quantified by comparison of their peak areas with those of calibration standards. The internal standard (methyl tricosanoate, Shanghai Aladdin Biochemical Technology Co., Ltd., Shanghai, China) is used to correct for extraction and analytical variability, ensuring accurate quantification.

#### 2.5.4 Biochemical parameters in hemolymph and hepatopancreas

The concentrations of triglyceride (TG), total cholesterol (T-CHO), high-density lipoprotein cholesterol (HDL-C), and low-density lipoprotein cholesterol (LDL-C) in hemolymph were measured using an automated biochemical analyzer (VITALAB SELECTRA Junior Pros, Vital Scientific B. V., Dieren, Gelderland, Netherlands). Aspartate aminotransferase (AST; C010-1-1) and alanine aminotransferase (ALT; C009-1-1) enzymatic activities, along with malondialdehyde (MDA; A003-1-2) concentrations, were quantified according to the manufacturer's protocols using commercial kits (Nanjing Jiancheng Bioengineering Institute, Nanjing, Jiangsu, China), and the absorbance and fluorescence were measured using a SpectraMax M2 microplate reader (Molecular Devices, LLC, San Jose, CA, USA).

Partial hepatopancreas samples from each treatment were thoroughly homogenized on ice in 0.9% saline solution using an IKA T25 digital Ultra-Turrax homogenizer (T25 digital, IKA-Werke GmbH & Co. KG, Staufen, Baden-Wuerttemberg, Germany). The homogenates were then centrifuged at 1229 × *g* for 10 min at 4 °C. The supernatants were distributed in aliquots into 200-μL PCR tubes and cryopreserved at −80 °C for subsequent analytical procedures. Concentrations of total protein (TP; A045-2-2), TG (A110-1-1), T-CHO (A111-1-1), LDL-C (A113-1-1), and MDA, as well as the activity of total superoxide dismutase (T-SOD; A001-1-2), were determined using commercial assay kits (Nanjing Jiancheng Bioengineering Institute, Nanjing, Jiangsu, China) following the manufacturers’ instructions, and the absorbance and fluorescence were measured using a SpectraMax M2 microplate reader.

#### 2.5.5 One-carbon metabolism parameters in hemolymph and hepatopancreas

The concentrations of SAM (MK0028OA) and SAH (MK0022OA) in hemolymph and hepatopancreas were determined using enzyme-linked immunosorbent assay (ELISA) kits (Jiangsu Suzhengke Biotechnology Co., Ltd., Yancheng, Jiangsu, China) following the manufacturers’ instructions, and the absorbance was measured using a microplate reader (SpectraMax M2).

#### 2.5.6 Histological observation in hepatopancreas

Histological analysis of hepatopancreas samples was performed using hematoxylin-eosin (HE) and oil red O staining. For HE staining, paraffin-embedded samples were dried at 65 °C for 2 h, followed by dewaxing in xylene and rehydration through a graded ethanol series. Nuclei were stained with hematoxylin, differentiated in hydrochloric acid-ethanol, and blued with ammonia water. Cytoplasm was then stained with eosin, followed by dehydration, clearing, and mounting. For oil red O staining of frozen sections, samples were washed with 50% ethanol, stained with oil red O for 8 min, counterstained with hematoxylin for nuclei, and mounted using glycerin gelatin. Quantitative analysis of both staining methods was performed using ImageJ software (version 1.53t, National Institutes of Health, Bethesda, ML, USA), with three biological replicates randomly selected per treatment and five visual fields randomly selected from each replicate for quantitative calculation.

#### 2.5.7 Extraction of total DNA from gut microbiota and 16S rRNA identification

Intestinal samples of mud crab preserved in cryotubes were used for total DNA extraction via the cetyltrimethylammonium bromide (CTAB) method. Nucleic acid integrity was verified through 1% agarose gel separation, while quantitative assessment of DNA concentration and purity parameters was performed using a Nanodrop 2000 spectrophotometric system (Thermo Fisher Scientific Inc., Waltham, MA, USA). The V3–V4 hypervariable region of the 16S rRNA gene was amplified via PCR, with barcode-labeled specific primers and extracted DNA as the template. Amplified DNA fragments were isolated through 2% agarose gel separation, subsequently purified with a commercial gel extraction kit, and precisely quantified using the Qubit 4.0 fluorescence detection system (Thermo Fisher Scientific Inc., Waltham, MA, USA). The purified PCR products underwent high-throughput sequencing using the Illumina NextSeq2000 system (Illumina, Inc., San Diego, CA, USA), with sequencing services provided by Shanghai Majorbio Pharmaceutical Technology Co., Ltd. (Shanghai, China). Quality control of paired-end raw reads was performed using fastp (https://github.com/OpenGene/fastp). Sequence assembly was performed using FLASH (http://www.cbcb.umd.edu/software/flash). In the overlapping regions, a maximum mismatch rate of 0.2 was tolerated during the process. Operational taxonomic units (OTUs) were clustered at 97% sequence similarity using UPARSE v7.1 (http://drive5.com/uparse/), with chimeric sequences removed. To normalize sequencing depth across samples, all sequences were rarefied to the number of sequences in the smallest sample. Taxonomic annotation of OTUs was performed using the RDP classifier (http://rdp.cme.msu.edu/) against the Silva 16S rRNA gene database at a 70% confidence threshold, and community composition was summarized at multiple taxonomic levels.

Alpha diversity indices were calculated using mothur (http://www.mothur.org/wiki/Calculators), and differences between treatments were assessed via the Wilcoxon rank-sum test. Beta diversity and microbial community similarity were examined using principal coordinates analysis (PCoA) based on the Bray–Curtis distance, with PERMANOVA employed to test for significant differences between treatments. Functional prediction of the 16S rRNA gene sequences was conducted using BugBase (https://bugbase.cs.umn.edu/index.html). All 16S sequencing data were analyzed and visualized using the MajorBio Cloud Platform (https://cloud.majorbio.com) for bioinformatics analyses.

#### 2.5.8 RNA extraction, reverse transcription, and quantitative real-time PCR analysis

The quantitative real-time PCR was employed to evaluate the expression of genes related to lipid metabolism and one-carbon metabolism. Hepatopancreas samples preserved in RNAlater were used for total RNA extraction with TRIzol reagent (Nanjing Vazyme Biotech Co., Ltd., Nanjing, Jiangsu, China). The RNA quality and concentration were assessed via 1.2% agarose gel electrophoresis and a Nanodrop 2000 spectrophotometer. Samples with A_260/280_ and A_260/230_ ratios between 1.8 and 2.2 were considered suitable for subsequent analysis. Qualified RNA was reverse-transcribed into complementary DNA (cDNA) using the HiScript II Reverse Transcriptase kit (Nanjing Vazyme Biotech Co., Ltd., Nanjing, Jiangsu, China). Based on previous research and three algorithms (BestKeeper, geNorm, and NormFinder), β-actin was ultimately selected as the most stably expressed housekeeping gene (Tang et al., 2025). The stability values of β-actin, *ef1a*, and *gapdh* were shown in Fig. S1. Complete cDNA sequences of target genes were obtained from the National Center for Biotechnology Information (NCBI) and transcriptome sequencing databases. The primer sequences were computationally designed with Primer Premier 3.0 and commercially synthesizedby Tsingke Biotechnology Co., Ltd. (Beijing, China). The complete primer specifications and corresponding amplification efficiency (%) are recorded in Table S1.

The quantitative real-time PCR was performed with the LightCycler 96 thermal cycler (Roche Diagnostics International AG, Rotkreuz, Switzerland), which integrates real-time fluorescence detection. The protocol included an initial activation at 95 °C for 2 min, followed by 55 cycles of 95 °C for 10 s, 58 °C for 10 s, and 72 °C for 20 s. Relative gene expressions were calculated using the 2^−ΔΔCt^ method (Livak and Schmittgen, 2001), with the control treatment (0.00 + 1% SST) set as 1.

### 2.6 Statistical analysis

#### 2.6.1 Specific calculation formulas

The following formulas were used to calculate percent weight gain (PWG), specific growth rate (SGR), survival, feed efficiency (FE), and alpha diversity indices of the gut microbiota:

$$\text{PWG}\;\left(\%\right)=100\times \left(W_{2}\;–\;W_{1}\right)/W_{1}\text{;}$$

$$\text{SGR}\;\left(\%/d\right)=100\times \left(\ln\;W_{2}\;–\;\ln\;W_{1}\right)/t\text{;}$$

$$\text{Survival}\;\left(\%\right)=N_{2}/N_{1}\times 100\text{;}$$

$$\text{FE}=\left(W_{2}\;–\;W_{1}\right)/D_{i}\text{;}$$

$$S_{Chao1}=S_{obs}+n_{1}\left(n_{1}\;–\;1\right)/2\left(n_{2}+1\right)\text{;}$$

$$D_{Simpson}=\sum_{i=1}^{S_{obs}}n_{i}\left(n_{i}\;–\;1\right)/N\left(N\;–\;1\right)\text{;}$$

$$H_{Shannon}=–\sum_{i=1}^{S_{obs}}n_{i}/N\times \ln\;n_{i}/N\text{.}$$

In the above formulas, *W*_*1*_ represents the average initial weight (g); *W*_*2*_ represents the average final weight (g); *t* represents the total experimental days; *N*_*1*_ represents the initial number of crabs; *N*_*2*_ represents the final number of surviving crabs; and *Dᵢ* represents the total dry diet intake (g). For alpha diversity indices, *S*_*Chao1*_ represents the Chao1 index; *S*_*obs*_ represents the observed OTU count; *n*_*1*_ and *n*_*2*_ represent the number of OTUs containing only one and two sequences, respectively, *D*_*Simpson*_ represents the Simpson index; *n*_*i*_ represents the sequence count contained in the *i*-th OTU; *N* represents the total sequence count, and *H*_*Shannon*_ represents the Shannon index.

Fatty acid data were analyzed using Origin 2022 software (OriginLab Corporation, Northampton, MA, USA) for generating cluster heatmaps and performing principal component analysis (PCA). Experimental data were subjected to one-way ANOVA using SPSS 22.0 (SPSS Inc., Chicago, IL, USA), and data visualization was performed using Prism 8 (GraphPad Software, Inc., San Diego, CA, USA). Additionally, the online software ChiPlot (https://www.chiplot.online/) was used to visualize the correlations between lipid metabolism-related genes and the contents of EPA and DHA in the hepatopancreas and muscle. Briefly, the Spearman rank correlation coefficients among the genes were first calculated, followed by the Mantel test to determine the Mantel correlation coefficients and significance between the contents of EPA/DHA and the genes.

#### 2.6.2 Statistical analysis

Statistical analyses were conducted according to the following model:

$$X_{ij}=\mu +\alpha_{i}+\varepsilon_{ij}\text{,}$$

where *Xᵢⱼ* represents the dependent variable; *μ* represents the overall mean; *αᵢ* represents the treatment effect; and *εᵢⱼ* represents the random error. The experimental results are expressed as means and standard error of the mean (SEM) (*n* = 3). Normality distribution and variance homogeneity were verified before statistical evaluation. For intergroup comparisons, Tukey's post hoc analysis is applied with the significance level set at *P* < 0.05.

## 3 Results

### 3.1 Growth performance and feed utilization

**Fig. 1:**
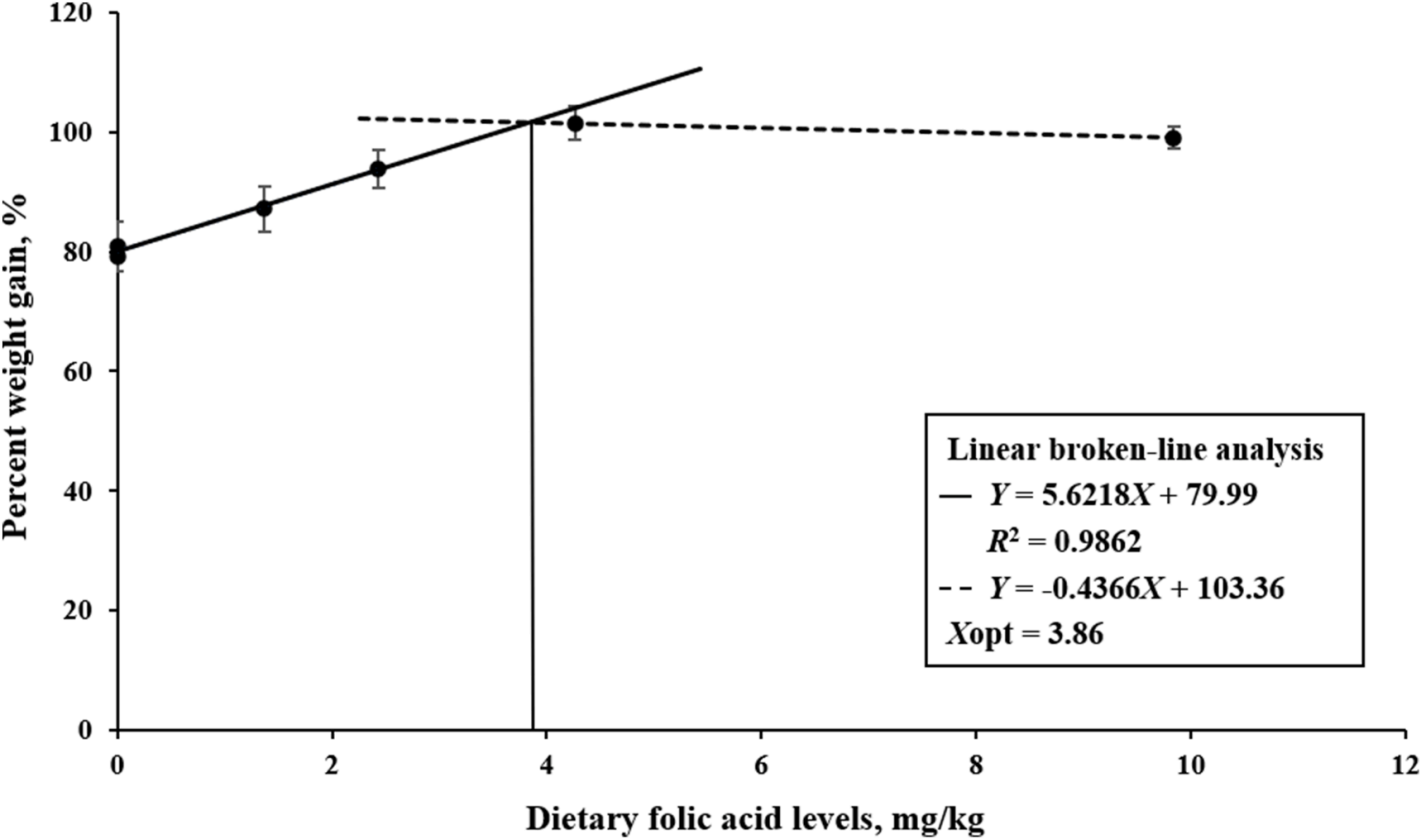
Two-slope broken-line regression analysis of the relationship between percent weight gain (PWG) and dietary folic acid (FA) levels. *X*opt represents the estimated optimal dietary folic acid level corresponding to the maximum PWG. The optimal dietary FA level was determined by solving for the intersection point between the regression equation of series 1 and the linear interpolation equation defined based on two points from series 2. Series 1 includes 0.00 + 1% SST, 0.00, 1.37 and 2.43 mg/kg FA treatments, while series 2 includes 4.26 and 9.84 mg/kg FA treatments. SST = succinylsulfathiazole.

The effects of dietary FA level on growth performance, survival, and feed utilization of juvenile mud crab are presented in Table 2. Compared with the 0.00 mg/kg FA and 0.00 + 1% SST treatments, dietary supplementation with 4.26 mg/kg FA improved final weight (FW), PWG, SGR, survival, and FE (*P* < 0.05). Throughout the entire study period, there was no significant difference in total feed intake among the treatments (*P* = 0.054). The two-slope broken-line regression of PWG against dietary FA levels estimated the optimal dietary FA level as 3.86 mg/kg for juvenile mud crab (Fig. 1).

**Table 2:** The effects of dietary folic acid level on growth performance of juvenile mud crab.

Items	Dietary folic acid levels, mg/kg						SEM	*P*-value
	0.00 + 1% SST	0.00	1.37	2.43	4.26	9.84		
Initial weight, g	15.23	15.17	15.35	15.18	15.18	15.16	0.024	0.150
Final weight, g	27.30^b^	27.45^b^	28.74^ab^	29.44^a^	30.59^a^	30.17^a^	0.339	0.001
Percent weight gain, %	79.21^c^	80.97^c^	87.24^bc^	93.91^ab^	101.50^a^	99.06^ab^	2.307	0.001
Specific growth rate, %/d	1.02^b^	1.02^b^	1.08^ab^	1.10^ab^	1.21^a^	1.19^ab^	0.022	0.012
Survival, %	42.06^c^	48.52^bc^	61.57^ab^	62.10^ab^	72.62^a^	69.05^a^	2.992	0.002
Feed efficiency	0.40^c^	0.45^bc^	0.51^bc^	0.53^b^	0.74^a^	0.65^a^	0.030	<0.001
Total feed intake, g	77.78	69.66	103.51	107.92	105.21	117.12	5.464	0.054

SST = succinylsulfathiazole; SEM = standard error of the mean.

The values in the same row with different superscripts are significantly different (*P* < 0.05), *n* = 3.

### 3.2 One-carbon metabolism

The effects of dietary FA level on one-carbon metabolism related parameters in the hemolymph and hepatopancreas of juvenile mud crab are shown in Table 3. Crabs fed diets containing 4.26 mg/kg FA exhibited higher hemolymph concentrations of SAM and SAH than those fed the other diets (*P* < 0.001), whereas crabs fed diets with 0.00 mg/kg FA supplemented 1% SST showed significantly reduced hemolymph SAH concentrations (*P* < 0.001). Similarly, 4.26 mg/kg FA increased SAM and SAH concentrations in the hepatopancreas (*P* < 0.001). In contrast, supplementation with 0.00 mg/kg FA supplemented and 0.00 mg/kg FA supplemented and 1% SST significantly decreased SAM and SAH concentrations in the hepatopancreas (*P* < 0.001).

**Table 3:** The effects of dietary folic acid level on one-carbon metabolism related parameters in the hemolymph and hepatopancreas of juvenile mud crab (pg/mL).

Items	Dietary folic acid levels, mg/kg						SEM	*P*-value
	0.00 + 1% SST	0.00	1.37	2.43	4.26	9.84		
**Hemolymph**								
SAM	233.21^c^	236.45^c^	238.00^bc^	243.01^ab^	248.75^a^	244.42^a^	1.355	<0.001
SAH	230.08^d^	240.61^c^	244.85^bc^	248.65^b^	254.45^a^	247.31^b^	1.900	<0.001
**Hepatopancreas**								
SAM	295.77^d^	319.09^c^	323.76^c^	331.25^b^	338.60^a^	333.75^ab^	3.444	<0.001
SAH	288.76^d^	320.40^b^	322.33^b^	330.55^a^	332.91^a^	309.96^c^	3.641	<0.001

SST = succinylsulfathiazole; SAM = S-adenosylmethionine; SAH = S-adenosylhomocysteine; SEM = standard error of the mean.

The values in the same row with different superscripts are significantly different (*P* < 0.05), *n* = 3.

**Fig. 2:**
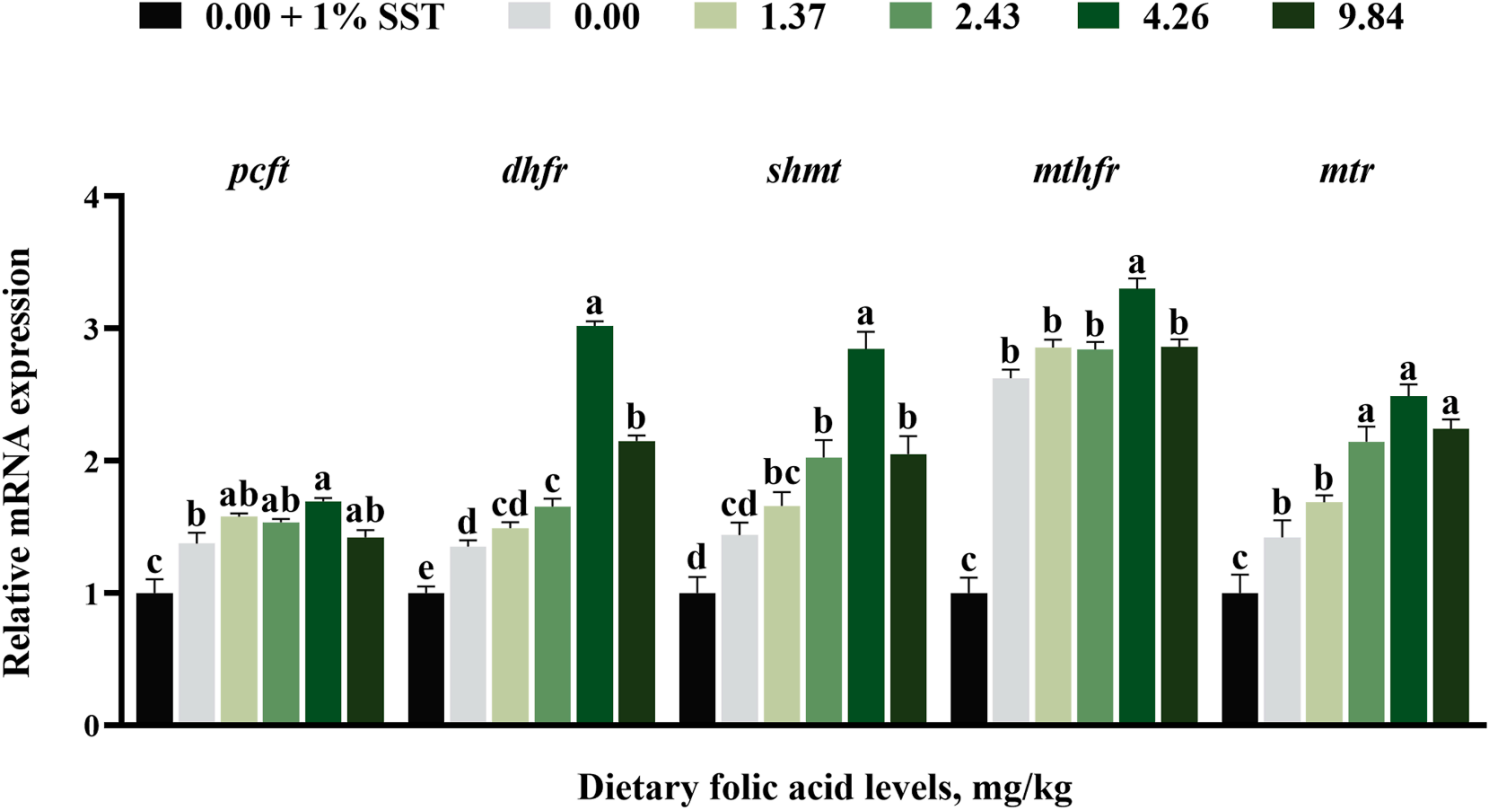
Effects of dietary folic acid level on the expression of one-carbon metabolism related genes in hepatopancreas of juvenile mud crab. Gene expression in the control treatment (0.00 + 1% SST) was normalized to 1. SST = succinylsulfathiazole. Different lowercase letters above columns represent significant differences among treatments at *P* < 0.05 (*n* = 3).

The effects of dietary FA level on the expression of one-carbon metabolism related genes in hepatopancreas of juvenile mud crab are shown in Fig. 2. Crabs fed diets containing 4.26 mg/kg FA exhibited significant upregulation of *dhfr*, *shmt*, and *mthfr* expressions in the hepatopancreas (*P* < 0.05; Fig. 2). As dietary FA increased from 0.00 to 4.26 mg/kg, the expressions of *pcft* and *mtr* were upregulated, followed by a decrease at higher FA level (*P* < 0.05). In addition, crabs fed diets with 0.00 mg/kg FA supplemented with 1% SST showed significant downregulation of *pcft*, *dhfr*, *mthfr*, and *mtr* in the hepatopancreas (*P* < 0.05).

### 3.3 Lipid metabolism

The effects of dietary FA level on lipid metabolism related parameters in the hemolymph and hepatopancreas of juvenile mud crab are presented in Table 4. Crabs fed diets containing 2.43 mg/kg FA exhibited significantly increased hemolymph concentrations of T-CHO and LDL-C, and also showed an increase in HDL-C (*P* < 0.001). In contrast, crabs fed diets with 0.00 mg/kg FA supplemented with 1% SST showed significantly decreased hemolymph HDL-C concentrations compared to those in the 0.00 mg/kg FA treatment (*P* < 0.001). Additionally, the 0.00 + 1% SST treatment resulted in decreased hemolymph T-CHO and LDL-C concentrations compared with other treatments. Dietary supplementation with 4.26 mg/kg FA significantly reduced hemolymph TG concentrations (*P* < 0.001).

**Table 4:** The effects of dietary folic acid level on lipid metabolism related parameters in the hemolymph and hepatopancreas of juvenile mud crab.

Items	Dietary folic acid levels, mg/kg						SEM	*P*-value
	0.00 + 1% SST	0.00	1.37	2.43	4.26	9.84		
**Hemolymph**								
T-CHO, mmol/L	0.12^c^	0.12^c^	0.17^b^	0.23^a^	0.16^b^	0.18^b^	0.010	<0.001
HDL-C, mmol/L	0.02^c^	0.06^b^	0.06^b^	0.10^a^	0.10^a^	0.09^ab^	0.007	<0.001
LDL-C, mmol/L	0.02^c^	0.02^bc^	0.02^bc^	0.04^a^	0.03^b^	0.03^b^	0.002	<0.001
TG, mmol/L	0.08^b^	0.10^a^	0.11^a^	0.12^a^	0.05^c^	0.07^b^	0.006	<0.001
**Hepatopancreas**								
T-CHO, mmol/gprotein	0.02^b^	0.03^b^	0.03^b^	0.06^a^	0.07^a^	0.06^a^	0.004	<0.001
LDL-C, mmol/gprotein	0.01^b^	0.01^b^	0.01^a^	0.01^a^	0.01^a^	0.01^a^	0.000	<0.001
TG, mmol/gprotein	0.03^b^	0.03^b^	0.04^b^	0.06^a^	0.06^a^	0.07^a^	0.004	<0.001

SST = succinylsulfathiazole; T-CHO = total cholesterol; HDL-C = high-density lipoprotein cholesterol; LDL-C = low-density lipoprotein cholesterol; TG = triglyceride; SEM = standard error of the mean.

The values in the same row with different superscripts are significantly different (*P* < 0.05), *n* = 3. Values shown in the table are rounded for presentation, whereas statistical significance is determined based on the original precise values.

**Fig. 3:**
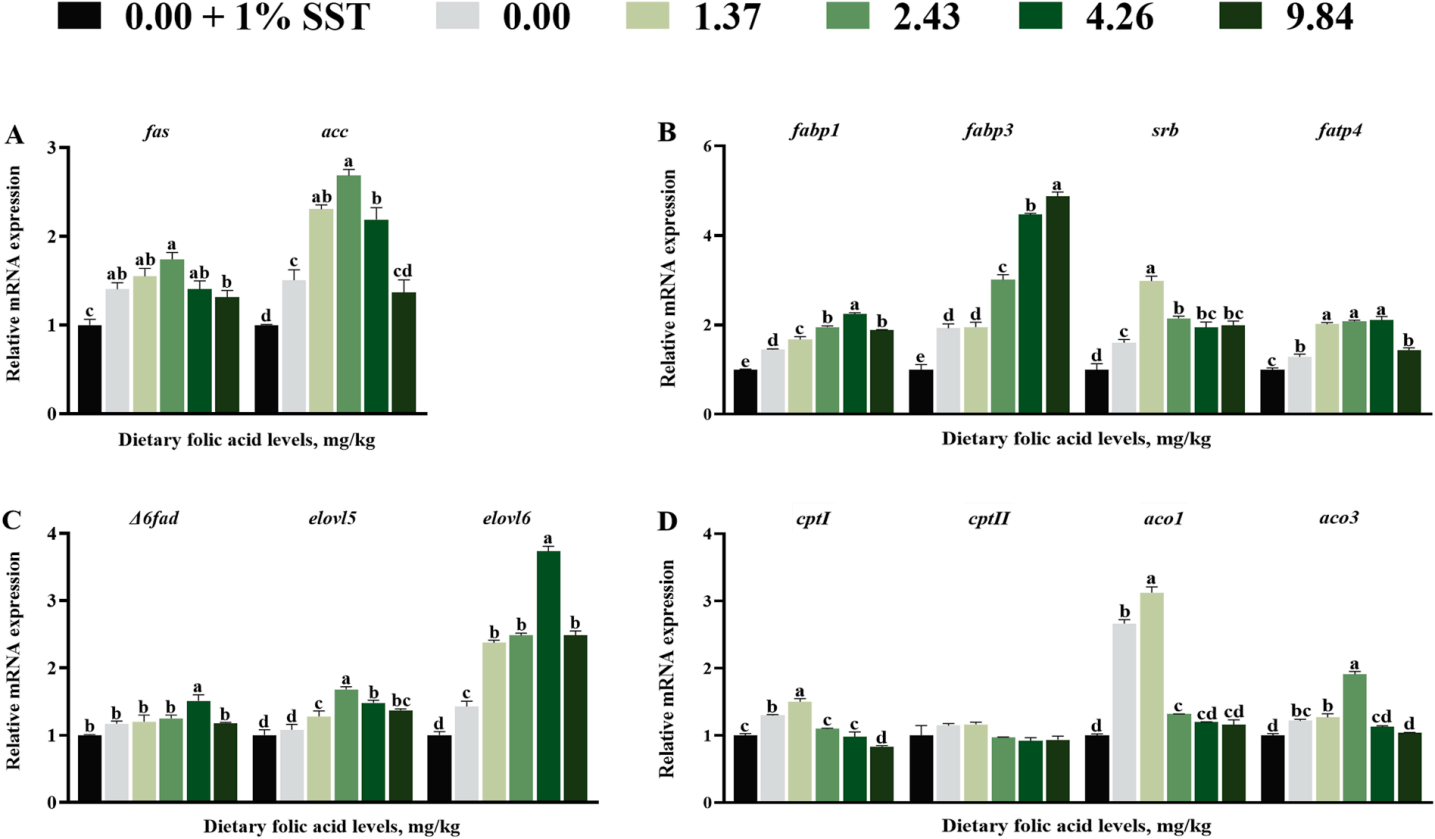
Effects of dietary folic acid level on the expression of genes associated with lipid metabolism in hepatopancreas of juvenile mud crab. (A) De novo fatty acid synthesis. (B) Lipid transport/uptake. (C) Fatty acid desaturation/elongation. (D) Fatty acid β-oxidation. Gene expression in the control treatment (0.00 + 1% SST) was normalized to 1. SST = succinylsulfathiazole. Different lowercase letters above columns represent significant differences among treatments at *P* < 0.05 (*n* = 3).

Compared with other treatments, dietary supplementation with 4.26 mg/kg FA increased T-CHO concentration in the hepatopancreas of juvenile mud crab, whereas 2.43 mg/kg FA enhanced LDL-C concentration (*P* < 0.001). Crabs fed 0.00 mg/kg FA supplemented with 1% SST exhibited decreased hepatopancreas T-CHO and LDL-C concentrations compared with other treatments (*P* < 0.001). Additionally, TG concentration in the hepatopancreas increased progressively with rising dietary FA level. The effects of dietary FA level on the expression of lipid metabolism related genes in hepatopancreas of juvenile mud crab are shown in Fig. 3. Dietary supplementation with 2.43 mg/kg FA upregulated expression of *fas* and *acc*, whereas 0.00 + 1% SST significantly downregulated expression of *fas* and *acc* (*P* < 0.05; Fig. 3A). Crabs fed 4.26 mg/kg FA exhibited significant upregulation of *fabp1*, while 9.84 mg/kg FA upregulated *fabp3* expression (*P* < 0.05). As dietary FA increased from 0.00 to 1.37 mg/kg, *srb* expression was significantly upregulated, followed by downregulation at higher FA level (*P* < 0.05). Moreover, 4.26 mg/kg FA upregulated *fatp4*, whereas 0.00 + 1% SST FA supplementation significantly downregulated *fabp1*, *fabp3*, *srb*, and *fatp4* (*P* < 0.05; Fig. 3B).

Dietary 4.26 mg/kg FA significantly upregulated the expressions of *Δ6 fad* and *elovl6*, whereas dietary 2.43 mg/kg FA significantly upregulated *elovl5* expression (*P* < 0.05). Notably, 0.00 + 1% SST FA supplementation significantly downregulated the expression of *elovl6* (*P* < 0.05; Fig. 3C). For fatty acid β-oxidation genes, 1.37 mg/kg FA significantly upregulated cptⅠ and *aco1*, and 2.43 mg/kg FA significantly upregulated *aco3* (*P* < 0.05). Dietary FA level had no significant effect on *cptⅡ*. Compared with the 0.00 mg/kg FA treatment, 0.00 + 1% SST FA supplementation significantly downregulated the expressions of *cptⅠ*, *aco1*, and *aco3* (*P* < 0.05; Fig. 3D).

### 3.4 Oil red O staining and HE staining in hepatopancreas

**Fig. 4:**
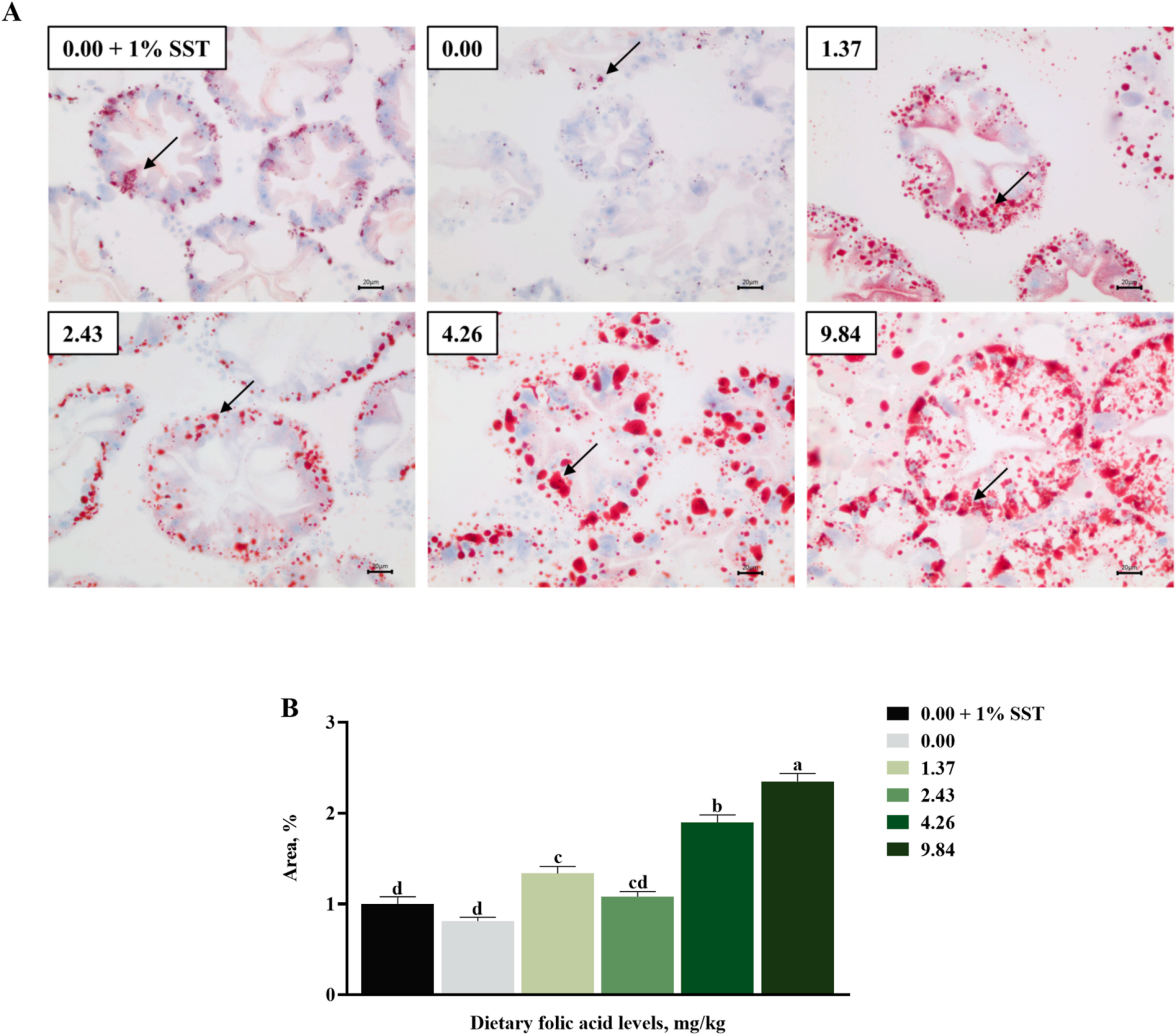
The oil red O staining in hepatopancreas of juvenile mud crab. (A) Oil red O-stained hepatopancreas sections (400 × magnification; scale bar = 20 μm). (B) Lipid droplet area (%). SST = succinylsulfathiazole. Different lowercase letters above columns represent significant differences among treatments at *P* < 0.05 (*n* = 3).

**Fig. 5:**
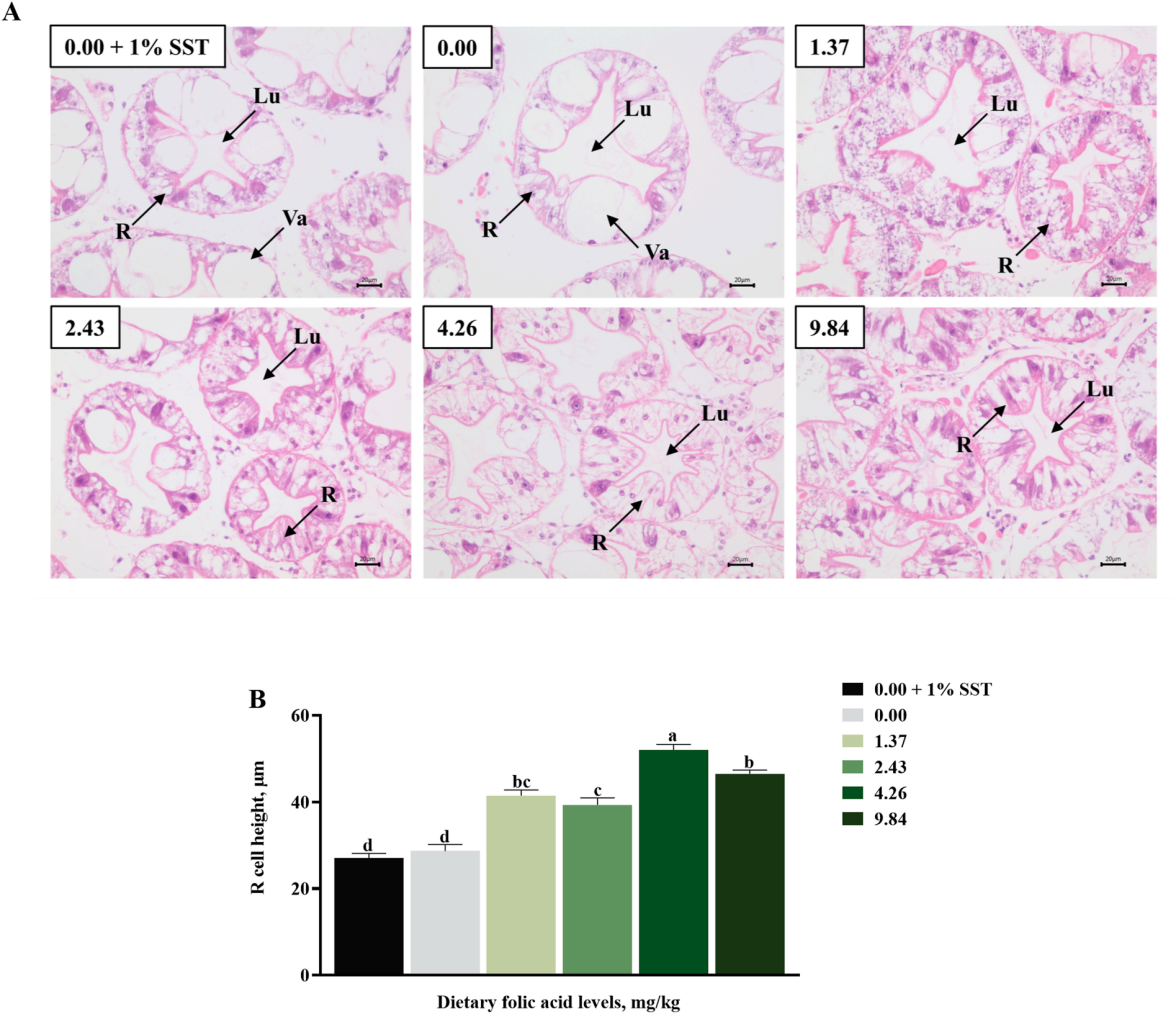
The hematoxylin-eosin (HE) staining in hepatopancreas of juvenile mud crab. (A) HE-stained hepatopancreas sections (400 × magnification; scale bar = 20 μm). (B) Resorptive cell height (R cell, μm). Lu = the lumen; R = resorptive cell; Va = vacuolization; SST = succinylsulfathiazole. Different lowercase letters above columns represent significant differences among treatments at *P* < 0.05 (*n* = 3).

Histological observations of the hepatopancreas of juvenile mud crab are presented in Fig. 4, Fig. 5. The oil red O staining revealed smaller lipid droplet areas in both the 0.00 mg/kg FA and 0.00 + 1% SST treatments (Fig. 4A). As dietary FA level increased from 0.00 to 9.84 mg/kg, the proportion of lipid droplet area in the hepatopancreas increased significantly (*P* < 0.05; Fig. 4B).

The HE staining showed that crabs fed 0.00 mg/kg FA and 0.00 mg/kg FA + 1% SST exhibited loosely arranged hepatopancreatic tubules, cytoplasmic vacuolization in some epithelial cells, and thinning of the tubule walls. In contrast, dietary supplementation with 4.26 and 9.84 mg/kg FA alleviated these histopathological alterations, resulting in more compact hepatopancreatic structures with clearly defined lumens (Fig. 5A). Furthermore, crabs fed 4.26 mg/kg FA showed a significant increase in the height of R cells within the hepatopancreatic tubules (*P* < 0.05; Fig. 5B).

### 3.5 The composition of fatty acids in hepatopancreas and muscle

**Fig. 6:**
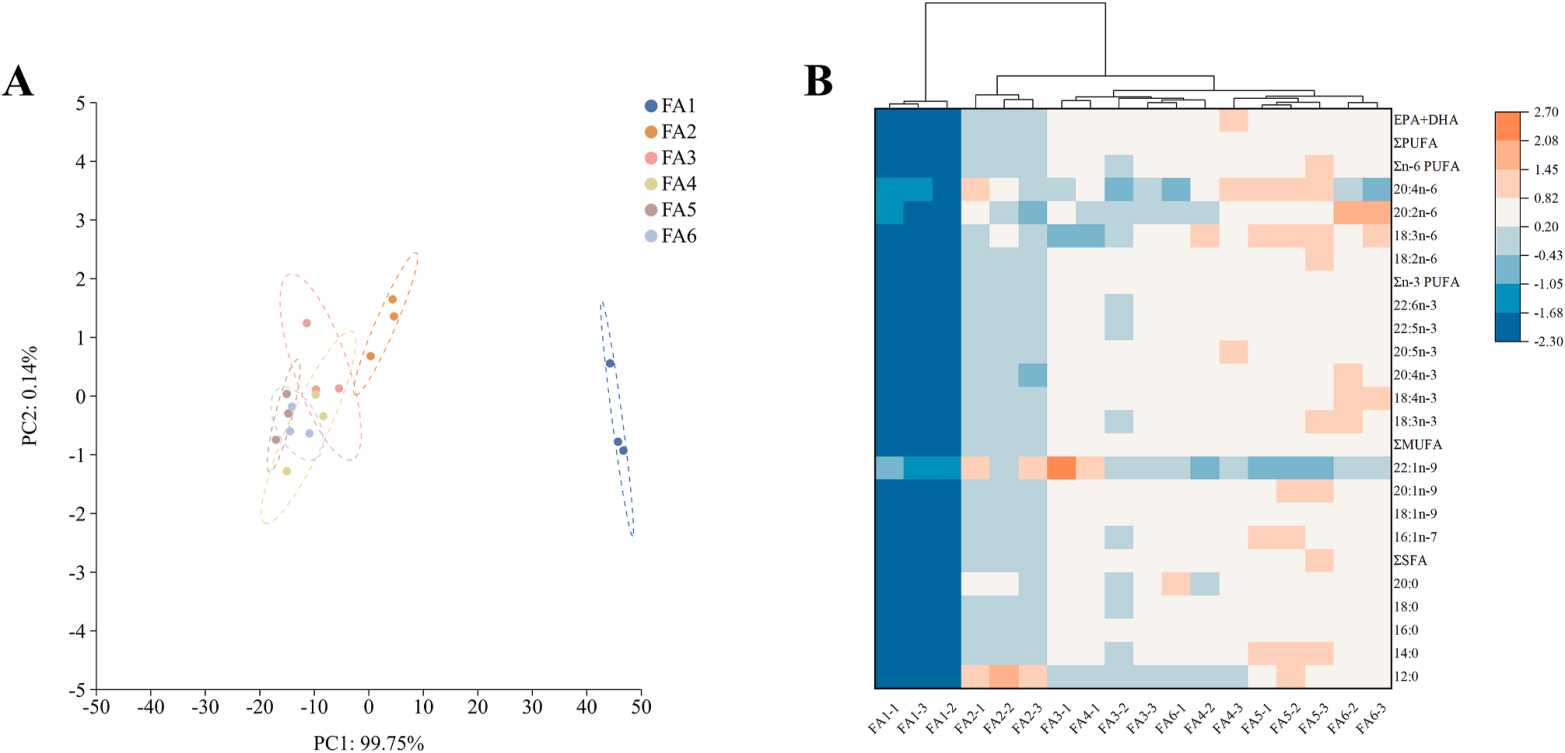
Effects of dietary folic acid (FA) level on the composition of fatty acids in hepatopancreas of juvenile mud crab. (A) Principal component (PC) analysis of hepatopancreatic fatty acids. (B) Cluster heatmap of hepatopancreatic fatty acids. Colors represent Z-score normalized abundances (red, higher; blue, lower). FA1, FA2, FA3, FA4, FA5, and FA6 correspond to the 0.00 + 1% SST, 0.00, 1.37, 2.43, 4.26, and 9.84 mg/kg folic acid treatments, respectively. SST = succinylsulfathiazole.

The effects of dietary FA level on the composition of fatty acids in the hepatopancreas of juvenile mud crab are presented in Table 5 and Fig. 6. Dietary supplementation with 4.26 mg/kg FA increased saturated fatty acids (SFA), monounsaturated fatty acids (MUFA), and polyunsaturated fatty acids (PUFA) contents in the hepatopancreas, while SST supplementation significantly reduced them. Similarly, 4.26 mg/kg FA enhanced n-3 PUFA and n-6 PUFA contents, whereas SST supplementation significantly reduced these levels. Furthermore, 4.26 mg/kg FA increased the concentrations of eicosapentaenoic acid (EPA; 20:5n-3) and docosahexaenoic acid (DHA; 22:6n-3) in the hepatopancreas.

**Table 5:** The effects of dietary folic acid level on the composition of fatty acids in hepatopancreas of juvenile mud crab (mg/g, dry matter).

Items	Dietary folic acid levels, mg/kg						SEM	*P*-value
	0.00 + 1% SST	0.00	1.37	2.43	4.26	9.84		
C12:0^1^	0.03^c^	0.08^a^	0.06^b^	0.06^b^	0.07^a^	0.06^b^	0.004	<0.001
C14:0	2.51^e^	5.05^d^	5.78^c^	5.99^bc^	6.61^a^	6.15^b^	0.329	<0.001
C16:0	15.17^c^	23.94^b^	26.68^a^	26.93^a^	27.85^a^	27.83^a^	1.090	<0.001
C18:0	5.59^c^	8.37^b^	9.00^a^	9.14^a^	9.51^a^	9.12^a^	0.325	<0.001
C20:0	0.36^b^	0.74^a^	0.76^a^	0.75^a^	0.80^a^	0.81^a^	0.038	<0.001
ΣSFA	23.66^d^	38.19^c^	42.27^b^	42.87^ab^	44.85^a^	43.97^ab^	1.780	<0.001
C16:1n-7	3.47^d^	7.47^c^	8.14^b^	8.64^ab^	8.98^a^	8.71^ab^	0.462	<0.001
C18:1n-9	21.38^d^	36.70^c^	40.71^b^	40.95^ab^	42.80^a^	41.72^ab^	1.801	<0.001
C20:1n-9	1.16^c^	2.09^b^	2.35^a^	2.35^a^	2.54^a^	2.38^a^	0.113	<0.001
C22:1n-9^1^	0.26^c^	0.85^a^	0.92^a^	0.68^ab^	0.37^bc^	0.63^ab^	0.077	0.049
ΣMUFA	26.27^c^	47.10^b^	52.13^a^	52.62^a^	54.68^a^	53.45^a^	2.406	<0.001
C18:3n-3	1.52^e^	3.26^d^	3.86^c^	3.97^bc^	4.41^a^	4.25^ab^	0.238	<0.001
C18:4n-3^1^	0.39^d^	1.02^c^	1.29^ab^	1.27^b^	1.36^ab^	1.45^a^	0.087	<0.001
C20:4n-3^1^	0.27^d^	0.52^c^	0.64^ab^	0.61^b^	0.67^a^	0.68^a^	0.035	<0.001
C20:5n-3	6.82^c^	11.68^b^	13.07^a^	13.60^a^	13.82^a^	13.64^a^	0.602	<0.001
C22:5n-3	1.15^c^	2.30^b^	2.51^a^	2.60^a^	2.62^a^	2.68^a^	0.130	<0.001
C22:6n-3	6.95^c^	11.91^b^	13.47^a^	13.89^a^	14.29^a^	13.87^a^	0.623	<0.001
Σn-3 PUFA	17.10^d^	30.69^c^	34.86^b^	35.93^ab^	37.16^a^	36.57^ab^	1.709	<0.001
C18:2n-6	7.91^d^	13.49^c^	15.72^b^	15.94^ab^	16.66^a^	16.41^ab^	0.745	<0.001
C18:3n-6^1^	0.30^b^	0.53^a^	0.50^a^	0.54^a^	0.64^a^	0.60^a^	0.028	<0.001
C20:2n-6^1^	0.66^b^	0.83^a^	0.83^a^	0.83^a^	0.87^a^	0.92^a^	0.022	0.001
C20:4n-6	1.51^c^	2.13^a^	1.85^b^	2.09^a^	2.21^a^	1.83^b^	0.061	<0.001
Σn-6 PUFA	10.37^d^	16.98^c^	18.90^b^	19.39^ab^	20.38^a^	19.76^ab^	0.832	<0.001
ΣPUFA	27.47^d^	47.67^c^	53.76^b^	55.32^ab^	57.54^a^	56.33^ab^	2.540	<0.001
EPA + DHA	13.78^c^	23.59^b^	26.55^a^	27.49^a^	28.11^a^	27.51^a^	1.223	<0.001

SST = succinylsulfathiazole; ΣSFA = total saturated fatty acids; ΣMUFA = total monounsaturated fatty acids; Σn-3 PUFA = total omega-3 polyunsaturated fatty acids; Σn-6 PUFA = total omega-6 polyunsaturated fatty acids; ΣPUFA = total polyunsaturated fatty acids; EPA = eicosapentaenoic acid; DHA = docosahexaenoic acid; SEM = standard error of the mean.

The values in the same row with different superscripts are significantly different (*P* < 0.05), *n* = 3.

1 Fatty acids present at trace levels (<1 mg/g dry matter).

The PCA revealed significant differences in hepatopancreatic fatty acids composition between the 0.00 + 1% SST treatment and other dietary treatments. Additionally, crabs fed 4.26 and 9.84 mg/kg FA exhibited significant differences compared with the 0.00 mg/kg FA treatment (Fig. 6A). Cluster heatmap analysis indicated that the 0.00 + 1% SST treatment had lower hepatopancreatic fatty acids contents, whereas crabs fed 4.26 mg/kg FA showed comparatively higher contents (Fig. 6B).

**Fig. 7:**
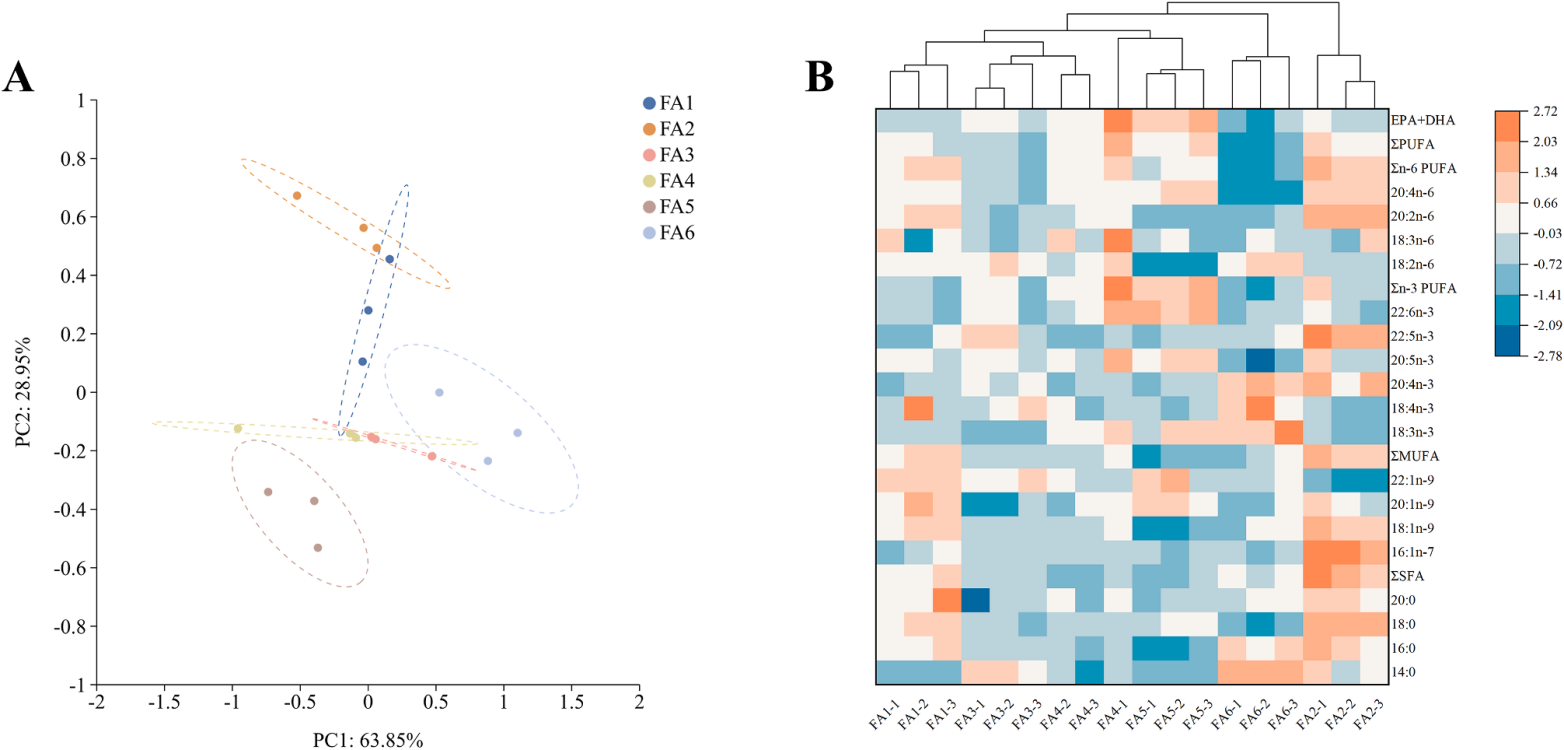
Effects of dietary folic acid (FA) level on the composition of fatty acids in muscle of juvenile mud crab. (A) Principal component (PC) analysis of muscle fatty acids. (B) Cluster heatmap of muscle fatty acids. Colors represent Z-score normalized abundances (red, higher; blue, lower). FA1, FA2, FA3, FA4, FA5, and FA6 correspond to the 0.00 + 1% SST, 0.00, 1.37, 2.43, 4.26, and 9.84 mg/kg folic acid treatments, respectively. SST = succinylsulfathiazole.

The effects of dietary FA level on the composition of fatty acids in muscle of juvenile mud crab are shown in Table 6 and Fig. 7. Dietary supplementation with 4.26 mg/kg FA decreased the contents of SFA and MUFA in the muscle of juvenile mud crab, while having no significant effect on PUFA. In addition, 4.26 mg/kg FA increased the contents of n-3 PUFA and the combined contents of EPA and DHA in muscle.

**Table 6:** The effects of dietary folic acid level on the composition of fatty acids in muscle of juvenile mud crab (mg/g dry matter).

Items	Dietary folic acid levels, mg/kg						SEM	*P*-value
	0.00 + 1% SST	0.00	1.37	2.43	4.26	9.84		
C14:0^1^	0.08^c^	0.10^b^	0.10^ab^	0.08^c^	0.08^c^	0.11^a^	0.004	<0.001
C16:0	2.92^ab^	3.00^a^	2.82^bc^	2.78^cd^	2.68^d^	2.98^a^	0.029	<0.001
C18:0	2.23^b^	2.33^a^	2.12^c^	2.11^c^	2.16^bc^	2.02^d^	0.025	<0.001
C20:0^1^	0.08	0.08	0.07	0.07	0.07	0.07	0.001	0.060
ΣSFA	5.31^b^	5.51^a^	5.10^c^	5.04^c^	4.99^c^	5.18^bc^	0.045	<0.001
C16:1n-7^1^	0.29^b^	0.36^a^	0.29^b^	0.29^b^	0.28^b^	0.28^b^	0.007	<0.001
C18:1n-9	3.09^ab^	3.19^a^	2.96^b^	2.98^b^	2.76^c^	2.98^b^	0.034	<0.001
C20:1n-9^1^	0.15^a^	0.15^a^	0.13^b^	0.14^ab^	0.15^a^	0.14^ab^	0.002	0.004
C22:1n-9^1^	0.10^a^	0.05^b^	0.09^a^	0.08^a^	0.10^a^	0.08^a^	0.005	<0.001
ΣMUFA	3.63^ab^	3.75^a^	3.47^b^	3.49^b^	3.29^c^	3.47^b^	0.037	<0.001
C18:3n-3^1^	0.15^bcd^	0.15^cd^	0.15^d^	0.16^ab^	0.16^abc^	0.17^a^	0.003	0.001
C18:4n-3^1^	0.02	0.02	0.02	0.02	0.02	0.03	0.001	0.077
C20:4n-3^1^	0.02^b^	0.03^a^	0.03^ab^	0.02^b^	0.02^b^	0.03^a^	0.001	0.002
C20:5n-3	4.29^a^	4.30^a^	4.31^a^	4.39^a^	4.40^a^	4.06^b^	0.033	0.006
C22:5n-3^1^	0.16^b^	0.20^a^	0.18^b^	0.16^b^	0.16^b^	0.17^b^	0.004	<0.001
C22:6n-3	3.58^b^	3.61^b^	3.62^b^	3.75^ab^	3.82^a^	3.59^b^	0.027	0.012
Σn-3 PUFA^6^	8.22^ab^	8.30^ab^	8.30^ab^	8.50^a^	8.58^a^	8.04^b^	0.053	0.018
C18:2n-6^1^	0.94^ab^	0.92^b^	0.95^ab^	0.94^ab^	0.84^c^	0.97^a^	0.010	<0.001
C18:3n-6^1^	0.05	0.05	0.05	0.06	0.05	0.05	0.001	0.500
C20:2n-6^1^	0.22^b^	0.25^a^	0.20^cd^	0.21^c^	0.18^e^	0.19^de^	0.005	<0.001
C20:4n-6	1.04^b^	1.13^a^	0.82^c^	1.02^b^	1.06^ab^	0.66^d^	0.040	<0.001
Σn-6 PUFA	2.26	2.34	2.01	2.22	2.14	1.87	0.039	<0.001
ΣPUFA	10.48^a^	10.65^a^	10.31^ab^	10.72^a^	10.72^a^	9.92^b^	0.079	0.002
EPA + DHA	7.86^ab^	7.90^ab^	7.93^ab^	8.14^a^	8.22^a^	7.65^b^	0.055	0.008

SST = succinylsulfathiazole; ΣSFA = total saturated fatty acids; ΣMUFA = total monounsaturated fatty acids; Σn-3 PUFA = total omega-3 polyunsaturated fatty acids; Σn-6 PUFA = total omega-6 polyunsaturated fatty acids; ΣPUFA = total polyunsaturated fatty acids; EPA = eicosapentaenoic acid; DHA = docosahexaenoic acid; SEM = standard error of the mean.

The values in the same row with different superscripts are significantly different (*P* < 0.05), *n* = 3.

1 Fatty acids present at trace levels (<1 mg/g dry matter).

The PCA revealed significant differences in muscle fatty acids composition between the 4.26 mg/kg FA treatment and both the 0.00 mg/kg FA and 0.00 + 1% SST treatments (Fig. 7A). Cluster heatmap analysis showed that 4.26 mg/kg FA decreased the contents of certain fatty acids in muscle relative to other treatments. Moreover, compared with the 0.00 mg/kg FA treatment, crabs fed diets with 0.00 mg/kg FA supplemented with 1% SST exhibited decreased contents of specific fatty acids, including 22:5n-3, 16:1n-7, and 20:4n-3 (Fig. 7B).

### 3.6 Correlation analysis

**Fig. 8:**
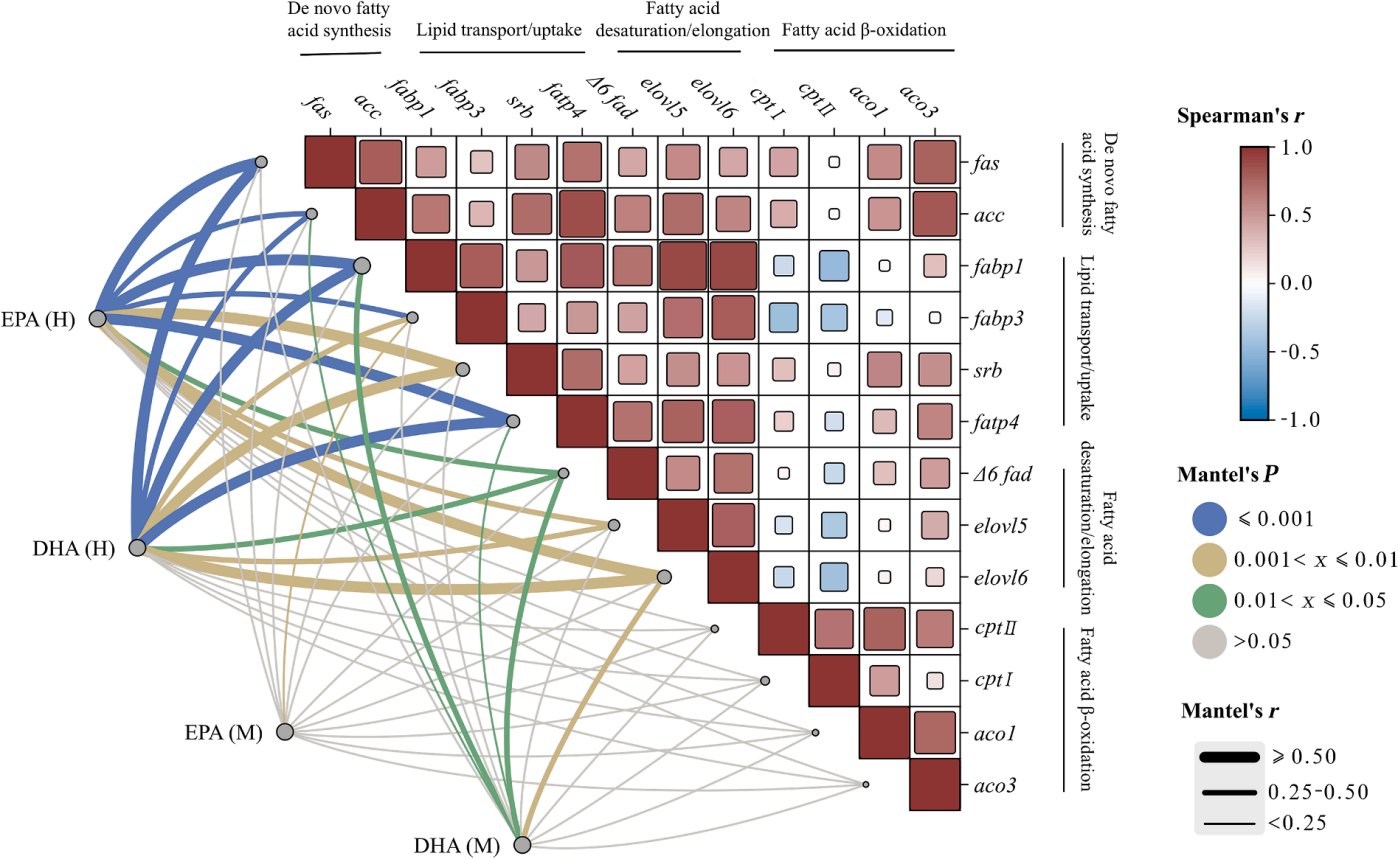
Mantel correlation analysis between lipid metabolism-related gene expression and EPA and DHA contents in the hepatopancreas and muscle. Line thickness represents the Mantel's *r* value, while line color indicates the significance level of the Mantel test. H represents hepatopancreas, and M represents muscle. EPA = eicosapentaenoic acid; DHA = docosahexaenoic acid.

The correlation analysis between lipid metabolism related genes and EPA and DHA contents in hepatopancreas and muscle are presented in Fig. 8. The results indicated that the content of EPA in hepatopancreas showed significant positive correlations with *fas* (*r* = 0.525), *acc* (*r* = 0.406), *fabp1* (*r* = 0.791), *fabp3* (*r* = 0.399), *srb* (*r* = 0.530), *fatp4* (*r* = 0.545), *Δ6 fad* (*r* = 0.268), *elovl5* (*r* = 0.427), and *elovl6* (*r* = 0.529) (*P* < 0.05). Similarly, the content of DHA in hepatopancreas showed significant positive correlations with *fas* (*r* = 0.521), *acc* (*r* = 0.406), *fabp1* (*r* = 0.787), *fabp3* (*r* = 0.387), *srb* (*r* = 0.530), *fatp4* (*r* = 0.552), *Δ6 fad* (*r* = 0.282), *elovl5* (*r* = 0.423), and *elovl6* (*r* = 0.542) (*P* < 0.05). In addition, both of them were negatively correlated with *cptⅠ*, *cptⅡ*, *aco1*, and *aco3*. In muscle, the content of EPA showed significant positive correlations with *fabp3* (*r* = 0.237), whereas DHA showed significant positive correlations with *acc* (*r* = 0.185), *fabp1* (*r* = 0.257), *fatp4* (*r* = 0.248), *Δ6 fad* (*r* = 0.288), and *elovl6* (*r* = 0.332) (*P* < 0.05).

### 3.7 Parameters related to hepatopancreas function and antioxidant capacity

The effects of dietary FA level on hepatopancreas function and antioxidant capacity-related parameters in juvenile mud crab are shown in Table 7. Crabs fed 4.26 mg/kg FA exhibited decreased activities of AST and ALT in hemolymph (*P* < 0.001). Dietary 4.26 mg/kg FA also significantly decreased the concentration of MDA in both hemolymph and hepatopancreas compared with other treatments (*P* < 0.001). Furthermore, 4.26 mg/kg FA significantly increased T-SOD activity in the hepatopancreas (*P* < 0.001).

**Table 7:** The effects of dietary folic acid level on hepatopancreas function and antioxidant capacity related parameters in juvenile mud crab.

Items	Dietary folic acid levels, mg/kg						SEM	*P*-value
	0.00 + 1% SST	0.00	1.37	2.43	4.26	9.84		
**Hemolymph**								
AST, U/L	4.78^a^	4.48^a^	4.22^a^	3.56^b^	2.63^c^	3.59^b^	0.179	<0.001
ALT, U/L	8.88^a^	8.40^a^	7.87^ab^	5.83^bc^	3.82^c^	4.40^c^	0.510	<0.001
MDA, nmol/mL	4.12^a^	3.98^a^	3.54^a^	3.50^a^	2.22^b^	3.43^a^	0.164	<0.001
**Hepatopancreas**								
MDA, nmol/mgprotein	0.67^a^	0.62^a^	0.51^b^	0.49^b^	0.38^c^	0.55^b^	0.023	<0.001
T-SOD, U/mgprotein	2.04^d^	2.60^c^	2.66^c^	2.71^c^	3.08^a^	2.86^b^	0.078	<0.001

SST = succinylsulfathiazole; AST = aspartate aminotransferase; ALT = alanine aminotransferase; MDA = malondialdehyde; T-SOD = total superoxide dismutase; SEM = standard error of the mean.

The values in the same row with different superscripts are significantly different (*P* < 0.05), *n* = 3.

### 3.8 16S rRNA analysis of gut microbiota

#### 3.8.1 16S rRNA sequencing and data quality

A total of 24 phyla, 44 classes, 100 orders, 172 families, 270 genera, and 333 species of microorganisms were detected across all treatments. Following quality control and paired-end sequence assembly, 1,066,499 sequences totaling 448,752,133 bases were obtained, with an average sequence length of 420 bp per sample. After data standardization, the average sequence coverage remained 99.09%, indicating high accuracy and reliability of the sequencing results. Rarefaction curve analysis showed that curves based on both the Sobs and Shannon indices plateaued for each sample, suggesting sufficient sequencing depth to capture the majority of microbial diversity (Fig. S2).

#### 3.8.2 Alpha diversity

Alpha diversity indices were calculated at the OTU levels (Table 8). The Chao1 index assessed community richness, while the Shannon and Simpson indices reflected community diversity. Crabs fed diets with 0.00 mg/kg FA and 1% SST showed increased Chao1 and Shannon indices and decreased Simpson index (*P* < 0.05). Similarly, dietary 4.26 mg/kg FA enhanced Chao1 (*P* < 0.001) and Shannon indices (*P* = 0.008), while reducing the Simpson index (*P* = 0.008) compared with 0.00 mg/kg FA treatment.

**Table 8:** Alpha diversity analysis of gut microbiota of juvenile mud crab.

Items	Dietary folic acid levels, mg/kg						SEM	*P*-value
	0.00 + 1% SST	0.00	1.37	2.43	4.26	9.84		
**Community richness**								
Chao1	164.02^a^	54.83^b^	83.55^b^	73.13^b^	97.12^b^	63.83^b^	9.331	<0.001
**Community diversity**								
Shannon	2.13^a^	1.00^b^	1.27^b^	1.42^b^	1.60^ab^	1.30^b^	0.102	0.008
Simpson	0.20^b^	0.53^a^	0.43^a^	0.35^ab^	0.33^ab^	0.37^ab^	0.029	0.008

SST = succinylsulfathiazole; SEM = standard error of the mean.

The values in the same row with different superscripts are significantly different (*P* < 0.05), *n* = 3.

#### 3.8.3 Beta diversity and OTU composition

**Fig. 9:**
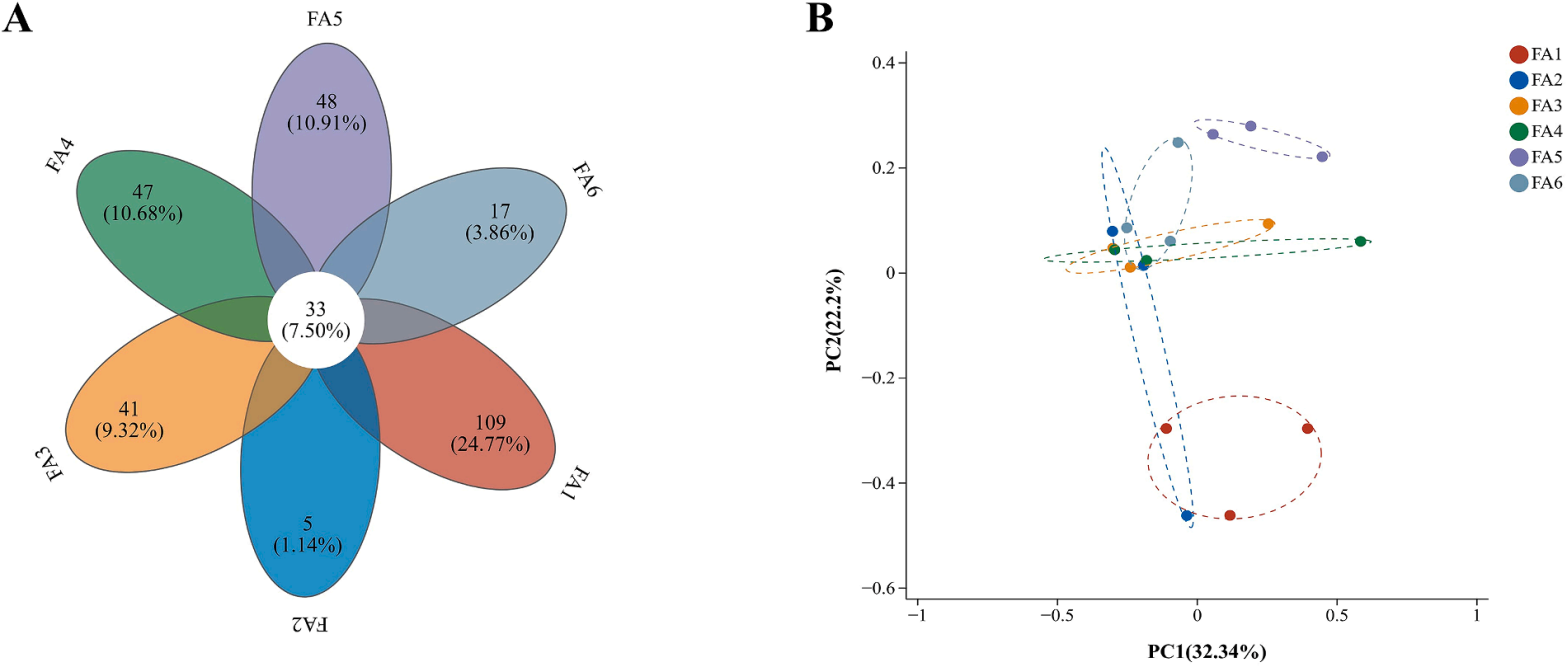
Effects of dietary folic acid (FA) level on the beta diversity and operational taxonomic unit (OTU) composition of gut microbiota in juvenile mud crab. (A) Venn diagram showing the number of OTUs in gut microbiota. (B) Principal coordinates analysis (PCoA) based on beta diversity. FA1, FA2, FA3, FA4, FA5, and FA6 correspond to the 0.00 + 1% SST, 0.00, 1.37, 2.43, 4.26, and 9.84 mg/kg folic acid treatments, respectively. SST = succinylsulfathiazole; PC = principal component.

A Venn diagram revealed 33 OTUs shared among all treatments. The 0.00 + 1% SST treatment had the highest number of unique OTUs (109), while the 0.00 mg/kg FA treatment had the lowest (5) (Fig. 9A). The PCoA based on beta diversity demonstrated significant separation between the 4.26 mg/kg FA treatment and other dietary treatments (Fig. 9B).

#### 3.8.4 Microbial composition at phylum and genus levels

**Fig. 10:**
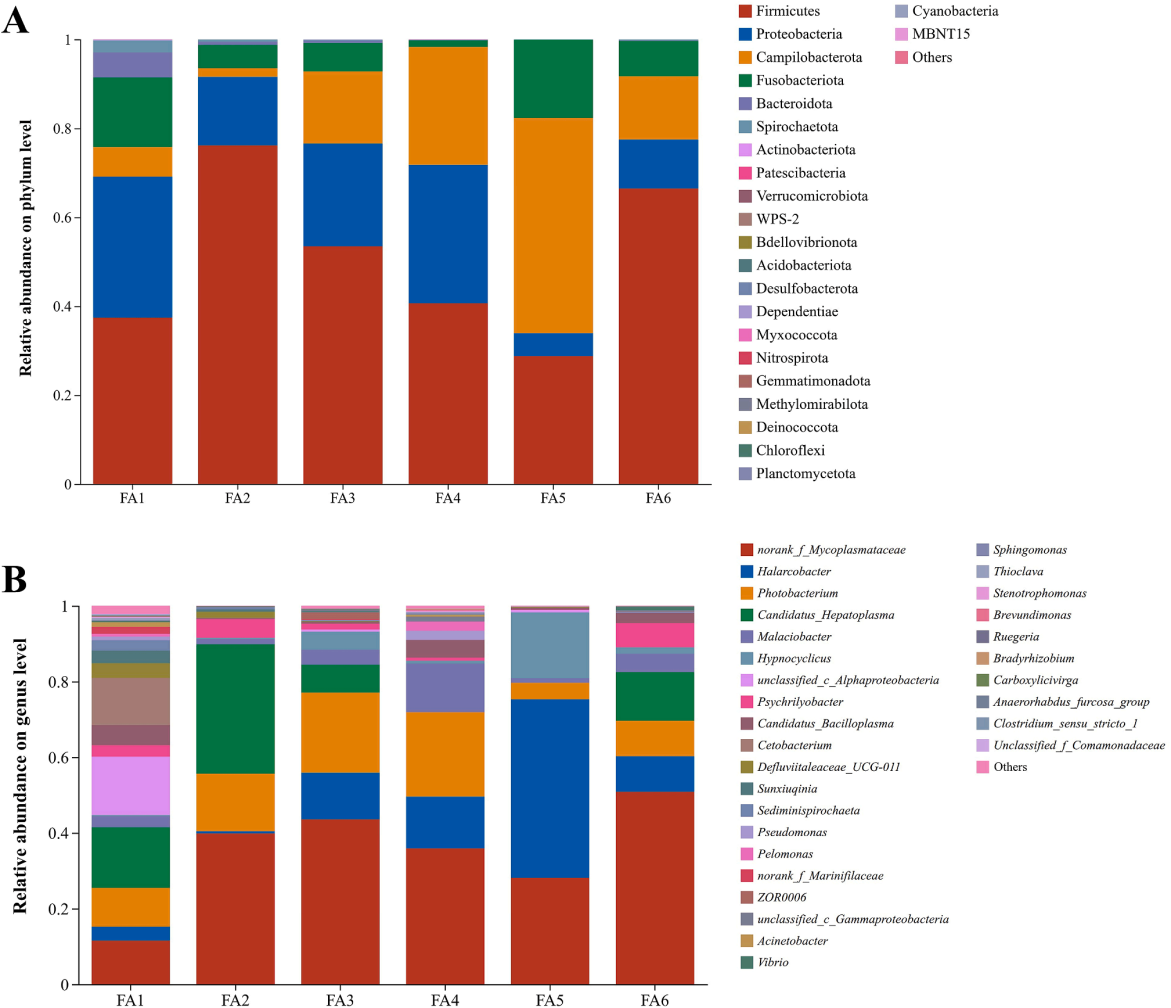
Effects of dietary folic acid (FA) level on the composition and relative abundance of gut microbiota in juvenile mud crab. (A) Relative abundance at the phylum level; (B) Relative abundance at the genus level. FA1, FA2, FA3, FA4, FA5, and FA6 correspond to the 0.00 + 1% SST, 0.00, 1.37, 2.43, 4.26, and 9.84 mg/kg FA treatments, respectively. SST = succinylsulfathiazole.

At the phylum level, Firmicutes, Proteobacteria, Campilobacterota, Fusobacteriota, and Bacteroidota were dominant in the gut microbiota (Fig. 10A). Compared with 0.00 mg/kg FA treatment, the 0.00 + 1% SST treatment increased the relative abundance of Proteobacteria, Campilobacterota, Fusobacteriota, and Bacteroidota, while decreasing Firmicutes. Dietary 4.26 mg/kg FA increased Campilobacterota and Fusobacteriota, but decreased Firmicutes and Proteobacteria.

At the genus level, *norank_f_Mycoplasmataceae*, *Halarcobacter*, *Photobacterium*, *Candidatus_Hepatoplasma*, and *Malaciobacter* were dominant (Fig. 10B). The 0.00 + 1% SST treatment increased *Halarcobacter* and *Malaciobacter*, while decreasing *norank_f_Mycoplasmataceae*, *Photobacterium*, and *Candidatus_Hepatoplasma*. Dietary 4.26 mg/kg FA increased *Halarcobacter* and *Hypnocyclicus*, while decreasing *Photobacterium*, *Candidatus_Hepatoplasma*, and *Malaciobacter*.

#### 3.8.5 Comparative analysis at the species level

**Fig. 11:**
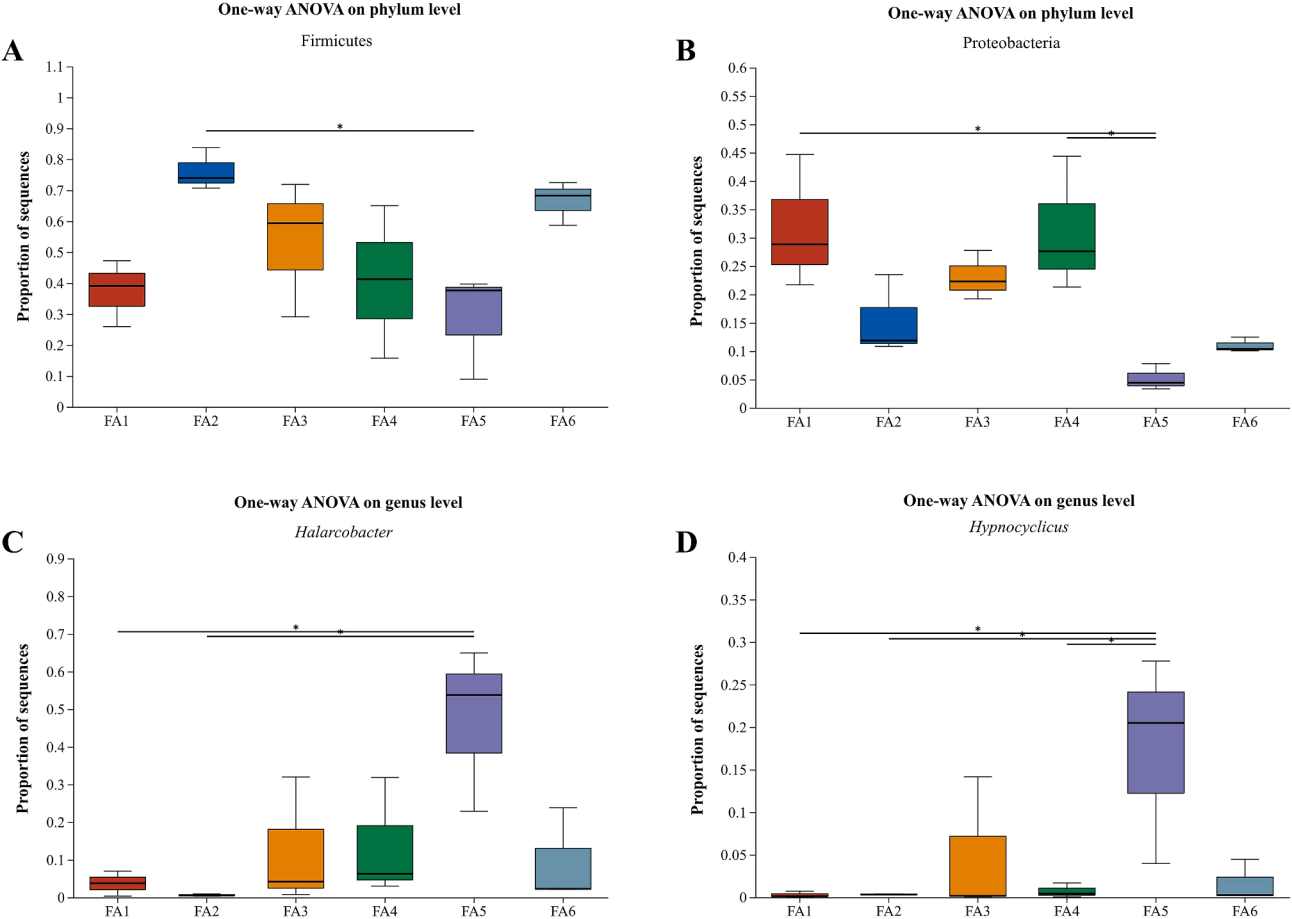
Effects of dietary folic acid (FA) level on differences in species composition of gut microbiota in juvenile mud crab. (A) Differences among Firmicutes. (B) Differences among Proteobacteria. (C) Differences among *Halarcobacter*. (D) Differences among *Hypnocyclicus*. FA1, FA2, FA3, FA4, FA5, and FA6 correspond to the 0.00 + 1% SST, 0.00, 1.37, 2.43, 4.26, and 9.84 mg/kg folic acid treatments, respectively. SST = succinylsulfathiazole.

The 4.26 mg/kg FA treatment significantly altered Firmicutes compared with the 0.00 mg/kg FA treatment and Proteobacteria compared with 0.00 + 1% SST and 2.43 mg/kg FA treatments (*P* < 0.05; Fig. 11A and B). At the genus level, *Halarcobacter* and *Hypnocyclicus* were significantly different in the 4.26 mg/kg FA treatment compared with both 0.00 + 1% SST and 0.00 mg/kg FA treatments (*P* < 0.05; Fig. 11C and D).

#### 3.8.6 Functional prediction

**Fig. 12:**
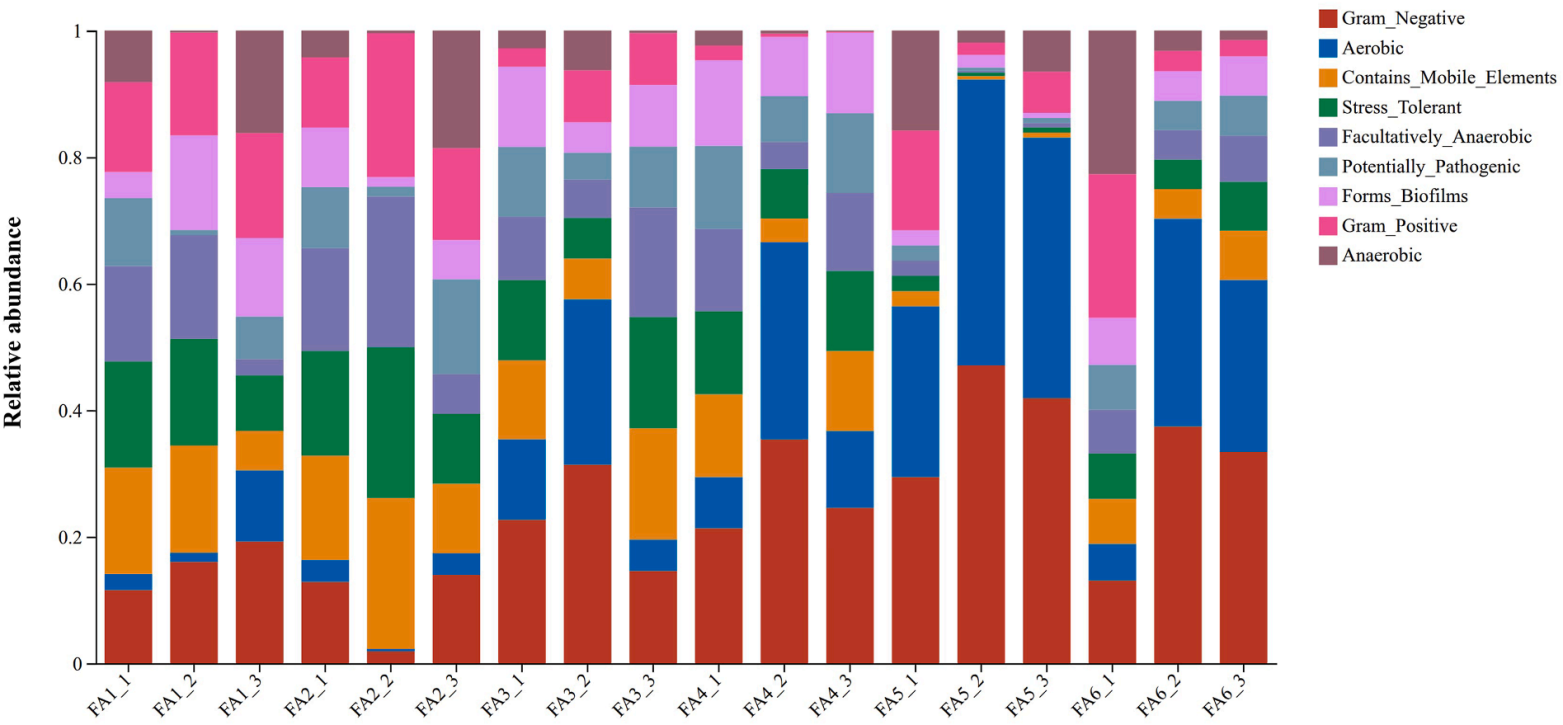
BugBase phenotype prediction analysis of gut microbiota in juvenile mud crab. FA1, FA2, FA3, FA4, FA5, and FA6 correspond to the 0.00 + 1% SST, 0.00, 1.37, 2.43, 4.26, and 9.84 mg/kg folic acid treatments, respectively. SST = succinylsulfathiazole.

The BugBase phenotype prediction indicated that gram-negative, aerobic, mobile element-containing, and oxidative stress-tolerant bacteria were predominant. Dietary 4.26 mg/kg FA increased the relative abundance of gram-negative and aerobic bacteria, while reducing oxidative stress-tolerant, anaerobic, and pathogenic bacteria (Fig. 12).

## 4 Discussion

As an indispensable micronutrient for crustaceans, FA is extensively utilized in diet formulation and modern aquaculture practices (Asaikkutti et al., 2016). Although numerous studies have described the beneficial influences of dietary FA on crustacean growth performance, the exact dietary requirements of this vitamin remain insufficiently defined for most aquatic species. Such uncertainty frequently leads to either inadequate or excessive supplementation in culture systems, both of which can impair growth and health, and in extreme cases, result in mortality (Liu et al., 2024). In the current research, crabs offered a FA-free diet (0.00 mg/kg) exhibited notably lower survival, whereas the inclusion of 4.26 mg/kg FA significantly enhanced survival relative to the unsupplemented treatment. This improvement is probably attributable to the essential role of FA in nucleic acid synthesis and cell proliferation (Cheng et al., 2024). As the major carrier of one-carbon units, THF, the metabolically active form of FA, participates in purine and pyrimidine biosynthetic pathways (Lawrence et al., 2011). During the juvenile developmental phase of the mud crab, rapid cell division increases the demand for FA. A deficiency in FA could therefore impair DNA replication accuracy, disrupt cellular functions, and reduce overall survival rates (Heyden et al., 2024). In agreement with results from giant freshwater prawn (*Macrobrachium rosenbergii*) and giant tiger shrimp (*Penaeus monodon*), dietary FA supplementation also promoted PWG and SGR in this study (Asaikkutti et al., 2016; Shiau and Huang, 2001). Juvenile mud crab receiving 4.26 mg/kg FA exhibited significantly higher FW, PWG, and SGR than those fed FA-deficient diets. Furthermore, enhanced FE in the FA-supplemented treatments indicated improved nutrient utilization, with the 4.26 mg/kg FA treatment producing higher FE compared with the 0.00 mg/kg FA treatment (Liu et al., 2023). Overall, these findings highlight that an appropriate dietary level of FA is indispensable for sustaining optimal growth, survival, and nutrient utilization in juvenile mud crab.

Succinylsulfathiazole is a long-acting sulfonamide antibiotic, and previous research has demonstrated that dietary inclusion of 1% SST markedly influences both the gut microbial composition and the FA biosynthetic capacity in animals (Beydoun et al., 2021). Owing to its structural similarity to para-aminobenzoic acid (PABA), a critical substrate for bacterial proliferation, SST competitively inhibits PABA by binding to dihydropteroate synthetase, thereby suppressing bacterial FA synthesis (Stokstad and Jukes, 1987). Moreover, SST exhibits minimal systemic toxicity, with its pharmacological activity largely confined to the gut, which makes it a widely adopted agent for evaluating FA nutritional requirements and for constructing animal deficiency models (Zhao et al., 2018). Notably, supplementation with 0.00 mg/kg FA and 1% SST produced no significant differences in FW, PWG, and SGR of juvenile mud crab compared with the 0.00 mg/kg FA treatment. However, a reduction in survival rate and feed efficiency was recorded under the 0.00 + 1% SST treatment. These observations suggest that juvenile mud crab have a limited capacity for de novo FA synthesis, which is insufficient to meet their metabolic demands during growth and development. Consequently, external FA provision through dietary supplementation remains essential for maintaining healthy growth performance (Asaikkutti et al., 2016). Based on broken-line regression analysis of PWG versus dietary FA concentrations, 3.86 mg/kg dietary FA was determined as the optimum nutritional requirement for juvenile mud crab. It is noteworthy that in this experiment, the optimal dietary FA requirement for juvenile mud crab was higher than the reported values for species like Chinese mitten crab (*E*. *sinensis*) and giant tiger shrimp (*P*. *monodon*). This discrepancy may be attributed to factors such as growth stages, feed formulations, environmental conditions, and evaluation criteria. Moreover, interspecific differences in endogenous FA synthesis capacity and utilization efficiency may further contribute to divergent requirements.

Growing evidence has demonstrated that FA plays a vital role in modulating lipid metabolism and supporting overall animal health (Chmurzynska et al., 2013; Fogacci et al., 2024). The liver or hepatopancreas is not only a central organ in lipid regulation but also a major site of FA metabolism, where numerous enzymes related to FA transformations have been identified (Christensen et al., 2015). Although decapod crustaceans, such as the mud crab, lack a true liver, their hepatopancreas fulfills analogous metabolic functions (Vogt, 2019). Previous research has confirmed that this organ serves as a pivotal regulator of lipid metabolism in mud crab (Chen et al., 2023; Zeng et al., 2024). Nevertheless, its involvement in FA metabolism has not yet been clarified. One-carbon metabolism constitutes a key biochemical pathway responsible for generating amino acids, nucleotides, and methyl donors, and it relies heavily on various folate derivatives acting as cofactors that mediate the transfer of single-carbon units between donors and acceptors (Petrova et al., 2023). This metabolic network encompasses two major interrelated cycles, the first being the FA cycle. Following intestinal absorption, dietary FA is transported to the liver via the proton-coupled folate transporter (PCFT), then sequentially reduced to dihydrofolate (DHF) and THF by dihydrofolate reductase (DHFR) using NADPH as a cofactor (Raimondi et al., 2019). In the present study, dietary supplementation with 4.26 mg/kg FA upregulated *pcft* expression and markedly upregulated *dhfr* expression in the hepatopancreas of juvenile mud crab. Serine hydroxymethyltransferase (SHMT), a pyridoxal phosphate-dependent enzyme, catalyzes the reversible conversion of L-serine and THF to glycine and 5,10-methylenetetrahydrofolate (5,10-CH_2_-THF). This conversion is considered the critical initial step of the FA cycle (McBride et al., 2024). Subsequently, under the catalytic action of methylenetetrahydrofolate reductase (MTHFR), 5,10-CH_2_-THF is transformed into 5-methyltetrahydrofolate (5-mTHF), which serves as an essential methyl donor (Gomez et al., 2024). In this study, dietary 4.26 mg/kg FA also induced significant upregulation of *shmt* and *mthfr* expressions, underscoring their crucial roles in maintaining FA cycling and one-carbon metabolism. Collectively, these findings suggest that appropriate FA supplementation enhances the one-carbon metabolic capacity of the hepatopancreas, thereby facilitating efficient methyl group transfer and sustaining cellular metabolic homeostasis in juvenile mud crab.

The methionine cycle constitutes another essential branch of one-carbon metabolism. Within this pathway, methionine synthase (MTR) catalyzes the transfer of methyl groups from 5-mTHF to homocysteine (Hcy), generating methionine and regenerating THF, a pivotal reaction connecting the FA and methionine cycles (Froese et al., 2019). In the present study, as dietary FA level increased from 0.00 to 4.26 mg/kg, the expression of *mtr* was upregulated, followed by downregulation at higher FA levels. This pattern suggests that dietary FA at 4.26 mg/kg facilitates the conversion of Hcy to methionine, thereby reducing Hcy accumulation and mitigating its potential cytotoxic effects (Peng et al., 2018; Robinson et al., 2018). Because methionine serves as the precursor for SAM, an imbalance in its availability may disturb lipid metabolism (Wang et al., 2016). Additionally, previous work has shown that SAM supplementation restores hepatic glutathione and decreases lipid peroxidation, thereby attenuating liver fibrosis in mammals (Gasso et al., 1996). Consistent with these findings, crabs receiving 4.26 mg/kg FA exhibited higher SAM concentrations in both hemolymph and hepatopancreas, indicating that adequate FA intake supports methionine recycling and SAM-dependent methylation activity. S-adenosylhomocysteine, the demethylated product of SAM, functions as a precursor of Hcy and a potent inhibitor of methyltransferase reactions (Cueto et al., 2024). Excess SAH accumulation can impair methylation balance and alter epigenetic regulation, potentially leading to metabolic disorders (Liu et al., 2024). In this study, dietary 4.26 mg/kg FA increased SAH concentration in both hepatopancreas and hemolymph, reflecting enhanced turnover within the methionine cycle. In contrast, supplementation with SST significantly downregulated one-carbon metabolism-related genes (*pcft*, *dhfr*, *mthfr*, and *mtr*) and reduced SAM and SAH concentrations compared with other treatments. Taken together, these findings demonstrate that dietary 4.26 mg/kg FA effectively sustains the integrity of the FA and methionine cycles in juvenile mud crab, whereas SST supplementation compromises endogenous FA synthesis and weakens one-carbon metabolic capacity.

Previous studies have demonstrated that lipid metabolism is closely associated with the growth performance and health status of crustaceans. Lipids play essential roles in various physiological processes, including molting, gonadal development, and hormone synthesis, while also serving as critical sources of energy and nutrients (Ghazali et al., 2017; Li et al., 2023; Zhong et al., 2023). As a fundamental structural element of cellular membranes and a biochemical precursor to steroid hormones, cholesterol plays a crucial regulatory role in the lipid metabolic processes of crustaceans (Ma et al., 2024). Through HDL-C and LDL-C, cholesterol is transported among tissues and converted into metabolites at specific sites (Meng et al., 2023). Dietary supplementation with 2.43 mg/kg FA elevated hemolymph concentrations of T-CHO, LDL-C, and HDL-C in the experimental crabs, while analogous lipid accumulation patterns were detected in hepatopancreas. Moreover, dietary supplementation with 4.26 mg/kg FA increased the T-CHO concentration in the hepatopancreas, while the 2.43 mg/kg FA enhanced LDL-C concentration. Considering the growth performance results, these findings indicate that FA contributes to maintaining cholesterol homeostasis in both the hemolymph and hepatopancreas of juvenile mud crab (Leclerc et al., 2021). On one hand, the abundant methyl groups provided by FA may facilitate the conversion of cholesterol into bile acids or their analogues and their subsequent excretion, thereby synergizing with dietary cholesterol to maintain hepatopancreatic health. On the other hand, the methyl deficiency state caused by FA insufficiency might suppress the expression of proteins related to cholesterol esterification or reverse transport, consequently antagonizing high cholesterol intake and exacerbating lipid deposition (Cui et al., 2017). The results showed that that 4.26 mg/kg FA improved the growth performance of juvenile mud crab, which may be partially attributed to its maintenance of normal lipid metabolic flux in the presence of cholesterol (Buettner et al., 2010). However, as the experimental design aimed to determine the requirement of a single nutrient and didn't include a factorial crossover trial of FA and cholesterol, their interaction effects cannot be directly quantified from the current data. Future research should adopt a two-factor experimental design to systematically investigate the combined effects of different supplementation levels of FA and cholesterol, thereby enabling the precise formulation of composite nutritional strategies for crustaceans.

To further determine the optimal dietary FA level for lipid metabolism, oil red O staining of the hepatopancreas was performed. The lipid droplet area (%) increased significantly as dietary FA level increased from 0.00 to 9.84 mg/kg, whereas the 0.00 mg/kg FA and 0.00 + 1% SST mg/kg treatments exhibited smaller lipid droplet areas. Triglycerides, the primary lipid storage form in juvenile mud crab, directly reflect lipid metabolic status (Ciaramella et al., 2014). In the current study, TG concentration in the hepatopancreas increased with higher dietary FA level, while crabs fed 4.26 mg/kg FA exhibited a significant decrease in hemolymph TG concentration. These results suggest that dietary supplementation with 4.26 mg/kg FA facilitates TG accumulation in the hepatopancreas while reducing circulating TG concentration in juvenile mud crab. Overall, these findings indicate that appropriate FA supplementation can optimize lipid distribution and promote a balanced lipid metabolic state in juvenile mud crab.

Marine decapod crustaceans are an excellent source of unsaturated fatty acids, with their abundant omega-3 polyunsaturated fatty acids (n-3 PUFA) known to prevent cardiovascular and cerebrovascular diseases, protect brain function, and alleviate inflammatory responses (Mori, 2017; Nanda et al., 2021). Among them, EPA and DHA have attracted widespread attention due to their diverse physiological and clinical benefits for human health (Alijani et al., 2025). The hepatopancreas and muscle of the mud crab not only constitute the primary edible portions but also serve as major storage sites for various fatty acids (Zhang et al., 2023). The results of the present study showed that crabs fed diets containing 4.26 mg/kg FA exhibited increased contents of SFA, MUFA, and PUFA in the hepatopancreas, along with elevated EPA + DHA levels. In contrast, the 0.00 + 1% SST treatment significantly reduced the contents of SFA, MUFA, and PUFA, as well as n-3 PUFA and omega-6 polyunsaturated fatty acids (n-6 PUFA) in the hepatopancreas. In muscle, dietary supplementation with 4.26 mg/kg FA increased n-3 PUFA and EPA + DHA levels, while decreasing SFA and MUFA contents, without significantly affecting total PUFA content. In addition, according to the results of the Mantel test correlation analysis, the EPA and DHA contents in the hepatopancreas were significantly positively correlated with *fas*, *acc*, *fabp1*, *fabp3*, *srb*, *fatp4*, *Δ6fad*, *elovl5*, and *elovl6*, while negatively correlated with *cptⅠ*, *cptⅡ*, *aco1*, and *aco3*. In muscle, EPA content correlated positively with *fabp3*, whereas DHA showed positive correlations with *acc*, *fabp1*, *fatp4*, *Δ6 fad*, and *elovl6*. Collectively, these findings suggested that appropriate FA supplementation effectively modulates tissue-specific fatty acid composition, enhancing the deposition of beneficial n-3 PUFA (particularly EPA and DHA) and thereby improving the overall nutritional quality of juvenile mud crab (Yuan et al., 2020). It is worth noting that folic acid-mediated regulation of lipid metabolism gene expression and tissue-specific fatty acid deposition may be driven by intracellular SAM/SAH homeostasis and subsequent epigenetic modifications (Zhang et al., 2025). Future studies will include analysis of the methylation status of key genes to validate this regulatory pathway as a critical research direction.

During de novo lipogenesis (DNL), acetyl-CoA carboxylase (ACC) catalyzes the conversion of acetyl-CoA to malonyl-CoA, which is subsequently utilized by enzymes such as FAS for stepwise fatty acid synthesis (Fhu and Ali, 2020). In the present study, dietary supplementation with 2.43 mg/kg FA upregulated the expressions of *fas* and *acc* in the hepatopancreas of juvenile mud crab, whereas the 0.00 + 1% SST treatment downregulated their expressions. Fatty acid binding proteins (FABPs) play crucial roles in intracellular lipid transport and solubilization. FABP1 binds endogenous fatty acids, while FABP3, belonging to the same superfamily, exhibits similar lipid-binding and transport functions (Michler et al., 2024; Yabut et al., 2024). Dietary 4.26 mg/kg FA significantly upregulated *fabp1* expression, whereas 9.84 mg/kg FA significantly upregulated *fabp3* expression, suggesting differential binding preferences for fatty acids under varying FA levels (Michler et al., 2024). Fatty acid transport protein-4 (FATP4), with long-chain acyl-CoA synthetase activity, indirectly mediates fatty acid uptake via vectorial acylation, while SRB is essential for fatty acid absorption (Li et al., 2023; Zhou et al., 2024). In this study, FA tended to upregulate *srb* and *fatp4* compared with the 0.00 + 1% SST or 0.00 mg/kg FA treatments. However, the differences among FA-supplemented treatments were not significant, indicating minimal regulation of these transporters by FA. The Δ6 FAD introduces double bonds to modify fatty acid saturation, whereas elongases of very long-chain fatty acids (ELOVLs) extend shorter fatty acids into longer chains (Dong et al., 2020). Dietary 4.26 mg/kg FA significantly upregulated expressions of *Δ6 fad* and *elovl6*, while 2.43 mg/kg FA significantly upregulated *elovl5* expression, indicating enhanced utilization of medium-chain fatty acids and promotion of SFAs and MUFAs conversion to PUFAs. Fatty acid β-oxidation is primarily regulated by CPT, which converts fatty acyl-CoA to acyl-carnitine for mitochondrial entry, and by ACO, the rate-limiting enzyme for long-chain fatty acid oxidation (Heinecke et al., 2020; Morash et al., 2009). In this study, dietary 1.37 mg/kg FA significantly upregulated *cptⅠ* and *aco1* expressions, whereas 2.43 mg/kg FA significantly upregulated *aco3* expression. However, *cptⅡ* expression remained unaffected by dietary FA level. These results suggest that when dietary FA exceeds 2.43 mg/kg, juvenile mud crab preferentially suppresses fatty acid β-oxidation, thereby promoting lipid deposition in the hepatopancreas. Overall, these findings indicate that appropriate FA supplementation modulates fatty acid synthesis, transport, desaturation, elongation, and oxidation in a dose-dependent manner, collectively favoring fatty acid accumulation and enhanced lipid deposition in the hepatopancreas of juvenile mud crab.

Excessive lipid deposition can lead to pathological damage in the hepatopancreas and disrupt lipid metabolism, posing significant threats to the health of juvenile mud crab (Luo et al., 2021). The hepatopancreas is primarily composed of embryonic cells (E), resorptive cells (R), blister-like cells (B), and fibrillar cells (F), among which R cells predominate and serve as the main site for lipid deposition (Aaqillah-Amr et al., 2018). In the present study, dietary supplementation with 4.26 mg/kg FA significantly increased the height of R cells in hepatic tubules, indicating enhanced lipid absorption and storage capacity. In contrast, both the 0.00 + 1% SST treatment and the 0.00 mg/kg FA treatment showed loose hepatic tubule arrangement, cytoplasmic vacuolization in some epithelial cells, and thinning of tubule walls, whereas dietary 4.26 mg/kg FA effectively preserved the normal morphology of hepatic tubules. The AST and ALT are key biomarkers for evaluating liver damage and overall health status, while MDA and SOD serve as indicators of oxidative stress (Falconí et al., 2024; Tsikas, 2017). The present results demonstrated that 4.26 mg/kg FA supplementation reduced AST and ALT activities in hemolymph, increased T-SOD activity in the hepatopancreas, and significantly decreased MDA concentration in both hemolymph and hepatopancreas. These findings suggest that dietary 4.26 mg/kg FA protects hepatopancreas structure and functions in juvenile mud crab by enhancing lipid absorption capacity while mitigating oxidative stress and lipid peroxidation, thereby maintaining overall hepatic health (Zhao et al., 2025).

The FA plays a significant role in modulating the composition and function of animal gut microbiota. For instance, FA has been reported to inhibit abdominal adipocyte proliferation and differentiation in broilers by altering gut microbiota composition, thereby reducing fat deposition (Liu et al., 2023). Another study showed that FA alleviated stress-induced performance decline in laying hens by maintaining gut microbiota homeostasis, promoting SCFA production, and suppressing bile acid biosynthesis (Sun et al., 2025). These findings collectively suggest that FA can influence host lipid metabolism through microbiota modulation, although this mechanism has not yet been reported in crustaceans. In the present study, 16S rRNA sequencing identified 24 phyla, 44 classes, 100 orders, 172 families, 270 genera, and 333 species, yielding 1,066,499 sequences and 448,752,133 bases. Compared with the 0.00 mg/kg FA treatment, dietary supplementation with 4.26 mg/kg FA induced restructuring of the gut microbiota in juvenile mud crab, as evidenced by elevated Chao1 and Shannon indices and a reduced Simpson index. These results collectively indicate that dietary 4.26 mg/kg FA not only enhanced microbial richness and diversity but also a potential augmentation of the community's functional capacity for nutrient metabolism (Nie et al., 2025). The OTU analysis revealed 33 shared OTUs among treatments, with the 0.00 + 1% SST treatment exhibiting the highest number of unique OTUs (109) and the 0.00 mg/kg FA treatment the fewest (5). The broad-spectrum antibiotic SST creates an ecological niche vacancy by inhibiting the original dominant bacterial populations, thereby disrupting the competitive balance of the gut microbial community. This provides proliferative opportunities for originally rare, antibiotic-resistant opportunistic bacteria, leading to an increase in the number of observed unique OTUs. However, this is not a sign of health but rather an indication of dysbiosis, characterized by disordered community structure and reduced specificity, which reflects a decline in microecological stability and resilience (Beydoun et al., 2021). In stark contrast, in an undisturbed natural state (0.00 mg/kg FA), the gut community maintains a fiercely competitive yet stable equilibrium. Dominant populations prevail through competitive exclusion, particularly under conditions where basic nutrients are relatively limited. Such an environment favors the survival of a small number of highly adapted core bacterial groups, thereby maximally suppressing the growth of other bacteria. This results in the most conserved and highly specific community structure (Zhang et al., 2022). The PCoA results showed no significant differences in overall microbial composition among treatments except for the 4.26 mg/kg FA treatment. Therefore, a concentration of 4.26 mg/kg may represent a critical ecological tipping point, suggesting that FA's regulation of microorganisms is dose-dependent. In this experiment, reaching this concentration was sufficient for FA to act as a key cofactor, significantly promoting the growth of specific beneficial bacteria that rely on FA, thereby altering the overall balance of the microbial community (Qiao et al., 2020). In summary, these results collectively indicate that 4.26 mg/kg FA supplementation guide the gut community toward a more beneficial and stable structural transformation.

The dominant phyla in juvenile mud crab gut microbiota included Firmicutes, Proteobacteria, Campilobacterota, Fusobacteriota, and Bacteroidota, consistent with previous studies (Saqib et al., 2023). Notably, dietary 4.26 mg/kg FA increased the relative abundance of Campilobacterota and Fusobacteriota, while reducing Firmicutes and Proteobacteria, suggesting that FA modulates gut microbial structure to improve host health (Shi et al., 2024). Many genera within the Firmicutes phylum exhibit strong energy acquisition capabilities and can enhance the host's absorption of dietary lipids. The decline in their relative abundance suggests that FA may moderately suppress excessive intestinal lipid absorption by modulating microbial composition, thereby helping maintain lipid balance in juvenile mud crab (Wu et al., 2023). Simultaneously, the increased abundance of Campilobacterota and Fusobacteriota may alter the metabolism and recycling processes of bile acids or their analogs in the gut, further influencing lipid emulsification and absorption efficiency (Thandar et al., 2024). This microbial structural adjustment likely represents one of the key mechanisms through which FA improves host health by optimizing lipid uptake to prevent metabolic burden. At the genus level, *Halarcobacter*, a halophilic group capable of chitin degradation, and *Hypnocyclicus*, a thermophilic genus producing highly stable amylolytic enzymes, were enriched in the 4.26 mg/kg FA treatment. These changes likely enhance carbohydrate utilization and high-salinity adaptability of juvenile mud crab (Meunier et al., 2024; Roalkvam et al., 2015). In crustaceans, excess carbohydrates can be metabolically converted into fat storage. Improved carbohydrate utilization helps maintain blood glucose homeostasis, reducing abnormal lipid mobilization caused by energy fluctuations, which collectively optimizes energy distribution and lipid metabolic balance (Guo et al., 2025). Functional analysis using BugBase revealed seven microbial phenotypes, consisting of Gram positive, Gram negative, biofilm forming, pathogenic, mobile element containing, oxygen demand (aerobic, anaerobic, and facultatively anaerobic), and oxidative stress tolerant (Zeng et al., 2025). Dietary 4.26 mg/kg FA increased gram-negative and aerobic bacteria while reducing oxidative stress-tolerant, anaerobic, and pathogenic bacteria. The increase in aerobic bacteria is often accompanied by elevated oxidative metabolic levels, which can promote the aerobic production of metabolites such as SCFAs. Certain SCFAs, like butyrate, have been demonstrated to modulate host lipid metabolism gene expression (Zhao et al., 2024). On the other hand, the reduction of pathogenic bacteria and oxidative stress-resistant bacteria suggests decreased intestinal inflammation levels, thereby improving the lipid metabolic environment. Overall, these findings indicate that dietary 4.26 mg/kg FA enhances gut health in juvenile mud crab by promoting beneficial microbial populations, suppressing potentially harmful bacteria, and improving nutrient metabolism and environmental adaptability.

## 5 Conclusion

In conclusion, the two-slope broken-line regression of PWG against dietary FA level indicated that the estimated optimal dietary FA level requirement for juvenile mud crab was 3.86 mg/kg. Dietary supplementation with 4.26 mg/kg FA enhanced the growth performance of juvenile mud crab by increasing the activities of key one-carbon metabolic enzymes and upregulating the expressions of related genes, thereby improving lipid metabolism and overall nutritional value. In addition, 16S rRNA analysis revealed that dietary 4.26 mg/kg FA increased gut microbial richness and diversity, which likely contributed to improved nutritional metabolism and environmental adaptability. Collectively, these results indicate that juvenile mud crab possess limited endogenous FA synthesis capacity, highlighting the necessity of dietary supplementation with 3.86 mg/kg FA to meet their nutritional requirements during growth and development.

## Credit Author Statement

**Zheng Tang:** Writing – review & editing, Writing – original draft, Methodology, Investigation, Formal analysis, Data curation, Conceptualization. **Yao Deng:** Validation, Methodology, Investigation, Formal analysis. **Shichao Xie:** Software, Formal analysis, Conceptualization. **Wenhao Zhan:** Visualization, Resources, Formal analysis. **Hongyu Peng:** Visualization, Software, Formal analysis. **Yinqiu Tian:** Investigation, Formal analysis. **Min Jin:** Writing – review & editing, Supervision, Project administration, Funding acquisition. **Tiantian Xu:** Resources, Investigation. **Xishuai Cui:** Resources, Investigation. **Xiaoyue Li:** Methodology, Investigation. **Peng Sun:** Validation, Supervision, Methodology, Conceptualization. **Qicun Zhou:** Writing – review & editing, Validation, Supervision, Project administration, Methodology, Funding acquisition, Conceptualization.

## Declaration of competing interest

We declare that we have no financial and personal relationships with other people or organizations that can inappropriately influence our work, and there is no professional or other personal interest of any nature or kind in any product, service and/or company that could be construed as influencing the content of this paper.
