# Supplementary material for: Dietary folic acid coordinates growth and lipid metabolism through gut microbial and one-carbon networks in juvenile mud crab (Scylla paramamosain)
Source: Anim Nutr. 2026 Jul 7;26:570–90. doi: 10.1016/j.aninu.2026.01.019 (PMC13474649; doi:10.1016/j.aninu.2026.01.019)
Supplement: Supplementary file 1 [file mmc1.docx]

**Table S1** Primers for quantitative real-time PCR of juvenile mud crab.

| Name | Primer type | Sequence (5’-3’) | Genbank No. | Amplification efficiencies, % |
| --- | --- | --- | --- | --- |
| **Housekeeper gene** | | | | |
| β-Actin | Sense | GCGGCAGTGGTCATCTCCT | KC795683.1 | 98.50 |
|  | Anti-sense | GCCCTTCCTCACGCTATCCT |  |  |
| *ef1a* | Sense | CTACAAGATTGGCGGCAT | JQ824130.1 | 102.21 |
|  | Anti-sense | GGGGGCAAAGTTCACGAC |  |  |
| *gapdh* | Sense | ACCTCACCAACTCCAACAC | (Jin et al., 2023) | 96.84 |
|  | Anti-sense | CATTCACAGCCACAACCT |  |  |
| **Lipid metabolism** | | | | |
| *acc* | Sense | CGAATGGAGTCCTCAATCTT | PRJNA634782 | 94.38 |
|  | Anti-sense | AGGTGACAGATTTCCGCTTC |  |  |
| *fas* | Sense | CATTCTGGGCATCAAGGA | HM217807.1 | 92.73 |
|  | Anti-sense | TGTTGGAGTCAGGGAGCA |  |  |
| *fabp1* | Sense | CACGCCATCCAACTCAAC | JQ824129.1 | 101.57 |
|  | Anti-sense | CATCACCATTCCCACACC |  |  |
| *fabp3* | Sense | TGAAGGCACTTGGTGTTGGA | PRJNA634782 | 99.85 |
|  | Anti-sense | ACTTCGGTGGTCTTCAGGGT |  |  |
| *fatp4* | Sense | ATTGGTGAGATTTGCCGCTA | PRJNA634782 | 97.22 |
|  | Anti-sense | ACAAAGCCGACTGCTCCTAC |  |  |
| *srb* | Sense | TATGCTGCTGACCCGTTC | LT797149.2 | 93.69 |
|  | Anti-sense | GGCACACCTAAGTTTGGC |  |  |
| *aco1* | Sense | TGAGGACAGATACGCTAACG | PRJNA634782 | 95.44 |
|  | Anti-sense | GGGTTGCCATCTTTTGTCAG |  |  |
| *aco3* | Sense | TTGATGATGTGGGCTGCTTT | PRJNA634782 | 105.59 |
|  | Anti-sense | TCTGTGCCAGTGGGTGAGGT |  |  |
| *cptⅠ* | Sense | CTTTTCCGTCGCCTTGTAGA | PRJNA634782 | 91.97 |
|  | Anti-sense | ATTGAGCAGCCGACCCTTAT |  |  |
| *cptⅡ* | Sense | GCACAACTCCCAAGTCATTC | PRJNA634782 | 103.82 |
|  | Anti-sense | AAAATGTCTGGCATAGGGGA |  |  |
| *Δ6 fad* | Sense | TAATCCTGACTGGGGACTGT | KU975033.1 | 100.85 |
|  | Anti-sense | AAGACTGGGTAGAGGCTGTT |  |  |
| *elovl5* | Sense | TGGGCACAGCACTTTCTTCG | PRJNA634782 | 99.23 |
|  | Anti-sense | TGACCTCCAATCCACCACAT |  |  |
| *elovl6* | Sense | TTCTCTTCGCTCGCTTCT | MF784574.1 | 104.11 |
|  | Anti-sense | ACCCTTGCTACCTTTCCC |  |  |
| **One-carbon metabolism** | | | | |
| *pcft* | Sense | AAGCCTGCAAGGGCAGAATA | PV232934 | 103.43 |
|  | Anti-sense | GAACACAGCTCCCAGTTCGT |  |  |
| *dhfr* | Sense | ATGGGGCTTCGTCTCAACAT | PV232933 | 105.09 |
|  | Anti-sense | GGATTCCCAGGTCTTGCGTC |  |  |
| *shmt* | Sense | TTGCTGGTGCCAGTGCTTAT | PV232935 | 94.61 |
|  | Anti-sense | AAGCCTGATCTGGGTCCTCT |  |  |
| *mthfr* | Sense | GCTGCCAATCACAAGCGTAG | PV232932 | 90.97 |
|  | Anti-sense | AAGCAGGAGATTCGGAGCTG |  |  |
| *mtr* | Sense | GCCGTATGGTCACAGGAAGAT | PV344628 | 96.88 |
|  | Anti-sense | GCTGTTGGTTGCCATTTGGA |  |  |

**Table S2** Abbreviations and full names of genes and proteins in this study.

| Abbreviations | Full names |
| --- | --- |
| **Proteins** |  |
| ACO | Acyl-CoA oxidase |
| CPT | Carnitine palmitoyl transferase |
| **Genes** |  |
| *acc* | Acetyl coenzyme A carboxylase |
| *aco1* | Acyl-CoA oxidase-1 |
| *aco3* | Acyl-CoA oxidase-3 |
| *cptⅠ* | Carnitine palmitoyltransferase Ⅰ |
| *cptⅡ* | Carnitine palmitoyltransferase Ⅱ |
| *dhfr* | Dihydrofolate reductase |
| *ef1a* | Eukaryotic translation elongation factor 1 alpha |
| *elovl5* | Elongase of very long-chain fatty acids |
| *elovl6* | Elongase of very long-chain fatty acids |
| *fabp1* | Fatty acid binding protein-1 |
| *fabp3* | Fatty acid binding protein-3 |
| *fas* | Fatty acid synthase |
| *fatp4* | Fatty acid transport protein-4 |
| *gapdh* | Glyceraldehyde-3-phosphate dehydrogenase |
| *mthfr* | 5,10-methylenetetrahydrofolate reductase |
| *mtr* | Methionine synthase |
| *pcft* | Proton-coupled folate transporter |
| *shmt* | Serine hydroxymethyltransferase |
| *srb* | Scavenger receptor b |
| *Δ6 fad* | Fatty acyl desaturase |

**Fig. S1** The stability of candidate reference genes was calculated using BestKeeper, geNorm and NormFinder algorithms. A lower stability value indicates greater stability.

**Fig. S2** Rarefaction curve analysis of gut microbiota diversity in juvenile mud crab. (A) Rarefaction curve based on the Sobs index. (B) Rarefaction curve based on the Shannon index. The *x*-axis represents the number of randomly selected sequencing reads, and the *y*-axis represents the number of observed species (Sobs) or the Shannon diversity index. FA1, FA2, FA3, FA4, FA5, and FA6 correspond to the 0.00 + 1% SST, 0.00, 1.37, 2.43, 4.26, and 9.84 mg/kg folic acid treatments, respectively. SST = succinylsulfathiazole; OTU = operational taxonomic unit.
